# Phytochemical-mediated green synthesis of selenium nanoparticles using *Catharanthus roseus* and their physicochemical characterization, biological evaluation, and molecular docking analysis

**DOI:** 10.1038/s41598-026-47919-3

**Published:** 2026-04-28

**Authors:** Abeer F. Desouky, Asmaa M. Fahim, Ayda K. Kelany, Marwa A. Kamel, Aly F. Mohamed, Mostafa M. Abo Elsoud, Sayeda A. Abdelhamid

**Affiliations:** 1 Department of Plant Biotechnology, National Research Centre, P.O. 12622, Dokki, Giza Egypt; 2 Department of Green Chemistry, National Research Centre, P.O. 12622, Dokki, Cairo Egypt; 3 Department of Genomic Medicine, Cairo University Hospitals, Cairo University, Cairo, 11599 Egypt; 4 Applied Science Research Center, Applied Science Private University, Amman, 11831 Jordan; 5 Department of Water Pollution Research, National Research Centre, P.O. 12622, Giza, Dokki Egypt; 6 Holding Company for Biological Products and Vaccines (VACSERA), P.O. 12622, Giza, Egypt; 7 Department of Microbial Biotechnology, National Research Centre, P.O. 12622, Dokki, Giza Egypt

**Keywords:** *Catharanthus roseus*, Selenium nanoparticles, Green nanotechnology, LC-MS/MS profiling, Molecular docking, Molecular dynamics, Biological activity, Biochemistry, Biological techniques, Biotechnology, Cancer, Chemical biology, Chemistry, Drug discovery

## Abstract

This study reports the green biosynthesis of bioactive selenium nanoparticles using *Catharanthus roseus* extract as a reducing and stabilizing agent. Liquid chromatography–electrospray ionization tandem mass spectrometry profiling identified key phytoconstituents, including riboflavin 5′-phosphate and chlorogenic acid, involved in the process. Characterization confirmed successful synthesis: ultraviolet–visible spectroscopy showed an absorption peak at 266 nm, high-resolution transmission electron microscopy revealed spherical particles ranging from 8.6 to 65.6 nm, zeta potential was − 3.55 mV, and X-ray diffraction confirmed crystallinity. Fourier-transform infrared spectroscopy indicated the involvement of phenolic and carboxyl groups. The biosynthesized selenium nanoparticles exhibited broad-spectrum antimicrobial activity, antiviral action against adenovirus (half-maximal inhibitory concentration (IC_50_) = 22.99 µg/mL), and potent cytotoxicity against HepG2 hepatocellular carcinoma cells (IC_50_= 1.5 µg/mL). Molecular docking and dynamics simulations suggested stable interactions between seleniumphytochemical complexes and biological targets. Overall, this work demonstrates that *C. roseus* provides a sustainable route to novel, multifunctional selenium nanoparticles with promising preliminary bioactivity, establishing a foundation for future therapeutic development and in vivo studies.

**Supplementary Information:**

The online version contains supplementary material available at 10.1038/s41598-026-47919-3.

## Introduction

Green nanotechnology has emerged as a promising approach for synthesizing functional nanoparticles using environmentally friendly methods, offering a sustainable alternative to traditional chemical and physical strategies^1–4^. Selenium (Se), a trace metalloid with diverse biological and technological applications, exhibits antioxidant, antimicrobial, antiviral, and anticancer properties. However, its toxicity at higher concentrations limits its direct therapeutic use^5,6^. Nanotechnology provides a strategy to mitigate selenium’s toxicity by producing selenium nanoparticles (SeNPs) with controlled size, morphology, and surface chemistry, while retaining or enhancing bioactivity^7^. Plant-mediated green synthesis of SeNPs has been widely explored, using extracts from species such as *Peltophorum pterocarpum*, *Capsicum annuum*, *Terminalia arjuna*, and fenugreek^8,9^. Recent studies published within the last five years have further expanded the application of plant extracts for the green synthesis of selenium nanoparticles, reporting SeNP production from diverse medicinal plants and evaluating their antimicrobial, antioxidant, and anticancer activities. For example, plant-mediated SeNPs synthesized using *Azadirachta indica*, *Moringa oleifera*, and *Allium sativum* extracts have demonstrated promising biological activities and improved biocompatibility compared with chemically synthesized counterparts^10,11^. Similarly, several investigations have explored SeNPs derived from plant metabolites for biomedical applications, including antimicrobial and anticancer therapies^7,12^. While these studies confirm the feasibility and biomedical potential of plant-mediated SeNP synthesis, most rely on crude plant extracts without detailed metabolomic characterization of the phytochemicals responsible for nanoparticle reduction and stabilization. Consequently, the mechanistic relationship between plant metabolite composition and the resulting nanoparticle physicochemical properties remains poorly understood. Moreover, only a limited number of studies integrate experimental biological evaluation with computational approaches to elucidate the molecular interactions underlying nanoparticle bioactivity. These studies demonstrate the feasibility of green synthesis; however, most focus primarily on nanoparticle formation, with limited investigation into how specific phytochemicals influence physicochemical properties and biological performance^13^. Therefore, a comprehensive understanding of how specific phytochemical constituents influence the reduction, stabilization, and biological functionality of selenium nanoparticles remains limited, representing a critical research gap in current green nanotechnology studies. *Catharanthus roseus* (L.) G. Don (Madagascar periwinkle) is an ideal model for bridging this gap. This pharmacologically important plant produces over 130 terpenoid indole alkaloids (TIAs), alongside flavonoids, phenolics, and quinones^14,15^. These metabolites contain functional groups (hydroxyl, amine, carbonyl) capable of reducing selenium salts and stabilizing nanoparticles, suggesting a dual role as reductants and capping agents. Despite extensive characterization of its phytochemistry^16,17^, the application of *C. roseus* for SeNP synthesis, with mechanistic insight into phytochemical-nanoparticle interactions, remains underexplored. The present study addresses this gap by systematically investigating *C. roseus*-mediated SeNP synthesis.  In this studies we Synthesize the SeNPs using a chemically characterized leaf extract of *C. roseus*, correlate specific phytochemical constituents with nanoparticle physicochemical properties (size, morphology, surface charge, crystallinity), and evaluate the bioactivity of the resulting SeNPs against microbial pathogens, viruses, and hepatocellular carcinoma cells. The novelty of this work extends beyond merely applying a new plant extract for SeNP synthesis. Unlike many recent green synthesis studies reported between 2021 and 2025, which primarily focus on nanoparticle formation and preliminary biological screening using crude plant extracts, the present study adopts an integrated phytochemical and mechanistic approach. In this work, LC–ESI–MS/MS metabolomic profiling was combined with comprehensive physicochemical characterization and computational analysis to identify phytochemicals potentially responsible for the reduction, capping, and stabilization of selenium nanoparticles (SeNPs). By correlating the phytochemical composition of *Catharanthus roseus* with nanoparticle properties and biological activity, this study provides deeper insight into plant-mediated SeNP biosynthesis and contributes to a more rational understanding of green nanomaterial design.

## Materials and methods

### Chemicals

Sodium selenite (Na_2_SeO_3_, analytical grade, ≥ 99%) was purchased from British Drug Houses LTD. Nutrient agar (NA), nutrient broth (NB), and potato dextrose agar (PDA) were from Biolife (microbiological grade). Potato dextrose broth (PDB) was from ARENA BioScien (microbiological grade). Potassium hydroxide (KOH, ≥ 85% purity) was from Alpha Chemika. Dimethyl sulfoxide (DMSO, ≥ 99.9%, molecular biology grade) and 3-(4,5-dimethylthiazol-2-yl)-2,5-diphenyltetrazolium bromide (MTT, ≥ 97%) were from Sigma-Aldrich. Vero cells (Vero, CCL-81) were obtained from the International Center for Advanced Research (ICTAR, Egypt). Minimum essential medium (MEM, sterile, cell culture grade), fetal bovine serum (FBS, heat-inactivated, cell culture grade), and streptomycin (cell culture grade) were from Biowest, France. 24-well plates and 96-well plates (tissue culture treated) were from SPL, Korea. THLE-2 and HepG2 cells were purchased from the Egyptian Holding Company for Vaccines and Sera (VACSERA) via the American Type Culture Collection (ATCC). All other chemicals and reagents used were of laboratory grade unless otherwise specified.

### Plant material collection and extract preparation

Plant material of *Catharanthus roseus* (L.) G. Don was obtained from the herbarium of the National Research Centre (NRC), Cairo, Egypt. The species was taxonomically authenticated by the herbarium staff, and a voucher specimen (Voucher No. PTBG1000042734; Specimen ID: 066191; Collector ID: 5458) is preserved in the institutional herbarium of NRC. The material was sourced from an institutional herbarium collection and was not collected from natural wild habitats or protected areas; therefore, no specific collection permits were required. All procedures complied with relevant institutional and national guidelines. The collected plant material was washed thoroughly with distilled water to remove dust and debris and then shade-dried at room temperature for 72 h. The dried material was finely cut, and 10 g was used for extract preparation. The material was extracted by heating in 200 mL of distilled water (1:20, w/v) using a water bath at 70 °C for 30 min. The extract was cooled to room temperature and filtered through Whatman No. 1 filter paper. The resulting clear filtrate was stored in sterile amber glass bottles at 4 °C and used for nanoparticle synthesis^18^.

### Identification and quantification of active compounds in plant extract using LC–MS/MS

The *C. roseus* extract was analyzed by liquid chromatography–electrospray ionization tandem mass spectrometry (LC–ESI–MS/MS) using an ExionLC AC system (USA) for chromatographic separation coupled with a SCIEX Triple Quad 5500 + MS/MS system (Singapore) equipped with an electrospray ionization (ESI) source. The ResPect spectral library was used in combination with MS-Dial software (version 4.70) for metabolite annotation. Chromatographic separation was performed using an Ascentis^®^ Express 90 Å C18 column (2.1 × 150 mm, 2.7 μm). The mobile phase consisted of two eluents: (A) 5 mM ammonium formate (pH 3) and (B) acetonitrile (LC grade). The gradient program was as follows: 5% B from 0 to 1 min, 5–100% B from 1 to 20 min, 100% B from 20 to 25 min, and re-equilibration to 5% B from 25.01 to 30 min. The flow rate was 0.3 mL/min with an injection volume of 5 µL. MS/MS analysis was conducted in positive ionization mode with a scan range of 100–1000 Da using the EMS-IDA-EPI acquisition method. The instrument parameters were set as follows: curtain gas 25 psi, ion spray voltage 5500 V, source temperature 500 °C, and ion source gases 1 and 2 at 45 psi. MS/MS spectra were acquired using a declustering potential of 80 V and collision energy of 35 eV^19^. Metabolite identification was achieved by comparing retention times, precursor ions (m/z), and characteristic MS/MS fragmentation patterns with those available in the ResPect database and published literature. B.

### Plant-mediated biosynthesis of SeNPs

The SeNPs were synthesized *via C. roseus* extract. Twenty mL of freshly prepared flower broth (extract) was mixed with 70 mL of a 10 mM aqueous solution of Na_2_SeO_3_ for preparing the reaction medium. This reaction medium was kept in an incubator under light at 36 °C for 1–10 days^18^.

### Ultraviolet-visible investigation

SeNPs production through *C. roseus* extract was noticed as a visual transformation after incubation. The UV/VIS Spectrophotometer (Shimadzu, UV-2401) has a wavelength range from 200 to 800 nm, allowing for further research through the optical characteristics of biosynthesized SeNPs^1^.

### High-resolution transmission electron microscopy

The high-resolution transmission electron microscope (HRTEM, JEM-2100 F) from Japan was used at 200 kV for confirming the formation of SeNPs *via C. roseus* extract, as well as investigating their size, dispersion, and selected area electron diffraction (SAED) pattern^30^.

#### Zeta potential analysis

The zeta potential, average diameter, and size distribution of SeNPs generated from *C. roseus* extract have been evaluated via a particle size analyzer (Nano-ZS, Malvern Instruments Ltd., UK). The samples had been sonicated for 10–15 min before being evaluated for zeta potential and the particle size distribution^31^.

#### Scanning electron microscopy

Scanning electron microscopy (SEM) and elemental analysis with EDX of the synthesized SeNPs generated through *C. roseus* extract using a scanning electron microscope (SEM-Quanta FEG250)^32^.

#### X-ray diffraction analysis

The SeNPs generated from *C. roseus* extract were investigated using X-ray diffraction (XRD) approaches (X-Pert, PRO, and Panalytical Netherlands)^33^. XRD was employed for examining the structural features of SeNPs produced at temperatures ranging from 10 °C to 80 °C. The average crystallite size (nm) was estimated from the XRD line broadening detection using Debye-Scherrer’s equation:

$$Crystallite\:size\:Dp = {{K\lambda } \mathord{\left/ {\vphantom {{K\lambda } \beta }} \right. \kern-\nulldelimiterspace} \beta }\:\cos \theta$$

Where Dp is the average crystallite size (nm), K = 0.94, λ = 1.54178 A°, β is the FWHM (full width at half maximum) of the XRD peak, and θ is the XRD peak position (half of 2θ).

#### Fourier transform infrared spectroscopy examination

The chemical structures of SeNPs produced by *C. roseus* extract were examined. The Fourier transform for infrared-attenuated total reflection spectroscopy (FTIR-ATR) is widely used for investigating adsorption and surface responses. FTIR-ATR evaluations have been performed employing a Bruker VERTEX 80 (Germany) in combination with a Platinum Diamond ATR that utilizes a diamond disk as an internal reflector in the 4000 –400 cm^−1^ range. The resolution is 4 cm^−1^, while the refractive index is 2.4^34^.

### Assessment of antimicrobial activity

#### Microbial strains and media

Four strains of bacteria, one fungus strain, and one yeast strain of considerable significance were used to evaluate the antimicrobial capacity of biosynthesized SeNPs from *C. roseus* extract, as well as the plant extract, which was used as a control. Two of the bacterial strains were considered to be Gram-positive (*S. aureus* NRRL B-313 and *B. subtilis* NRRL B-94), while the others were Gram-negative (*E. coli* NRC B-3703 and *P. aeruginosa* NRC B-32). The fungal strain was *A. niger* NRRL 599, and the yeast strain was *C. albicans* NRRL 477. Nutrient agar media was utilized in the current investigation for bacterial growth, while PDA was utilized to cultivate fungus and yeast strains. The medium was autoclaved at 121 °C for 20 min prior to being used for subculture. Solid media was then used for the agar-well diffusion assay.

#### Well diffusion assay

The method of well diffusion was used in evaluating the antimicrobial effects of SeNPs. Twenty mL of an agar medium (nutrient and PDA) was placed into sterile Petri dishes that had previously been seeded with a bacterial suspension containing LOG 0.9 CFU/mL of medium^35^. These microbes were cultivated and kept at 37 °C for 24 h for bacterial strains and at 30 °C for 48 h for fungal strains. To ensure a uniform microbial distribution, the inoculum suspensions were spread thoroughly over the agar plates by using a spreader. Using a sterile borer, a 6 mm diameter well was created in the inoculated media, and 0.2 mg/mL of biosynthesized SeNPs and controls were added. The plates were placed in the refrigerator at 4 °C for 2 h to allow the particles to diffuse. The presence of inhibition zones was evaluated as an indicator of antimicrobial activity. Ampicillin and Mycostatin have been utilized as reference antibiotics against evaluation bacteria and fungi, respectively. The procedure was carried out in triplicate, and the mean of the zones of inhibiting growth was determined.

### Determination of minimum inhibitory concentrations using the optical density assay

The optical density assay was used for evaluating the MIC effects of SeNPs from *C. roseus* extract on various test microbial organisms. Concentrations of 4.2–210 µg/mL had been added to a 3 mL nutrient broth and PDB culture for bacteria and fungi, respectively. The tubes were then inoculated with 50 µL of each adjusted fresh microbial strain. The growth and sterility controls included two blank nutrient broth tubes, one with and one without microbial inoculation. Incubate the bacterial strains in a shaking incubator at 120 rpm at 37 °C for 24 h, followed by the Candida isolate at 30 °C for 48 h. Growth of microbial strains was evaluated at 620 nm using a UV/VIS spectrophotometer (Shimadzu, UV-2401), and the results were displayed as inhibited growth percent. The first tube in the collection with no obvious development after the incubation period was determined as the MIC^36^.

### Determination of minimum bactericidal and fungicidal concentrations

The minimum bactericidal concentration (MBC) and minimum fungicidal concentration (MFC) of SeNPs from *C. roseus* extract against the test microbes have been determined using the Cos et al.^37^ method. Each tube contained 50 µL of fresh microbial strains. SeNPs concentrations ranged from 420 to 736.8 µg/mL in 3 mL of nutrient broth and PDB for bacteria and fungi, respectively. Bacterial strains were incubated at 37 °C for 24 h, while Candida isolates were incubated at 30 °C for 48 h. The MBC or MFC is recognized as the lowest concentration of SeNPs that caused no bacterial or fungal growth. To validate the results, the procedure was repeated in three independent collections.

### Assessment of antiviral activity

#### Cell culture and human adenovirus infectivity titer estimation

Vero cells (Vero, CCL-81) from the ICTAR were used to propagate and titrate human adenovirus type 40 (Adeno 40). Vero cells were cultured in Minimum Essential Medium with 10% fetal bovine serum, 100 U/mL penicillin, and 100 U/mL streptomycin at 37 °C in a humidified 5% CO_2_ incubator until they formed a confluent sheet. The virus seed stocks were serially diluted 10-fold in MEME medium containing 2% fetal calf serum and 1% antibiotic/antimycotic. Virus dilutions of 0.1 mL/well were dispersed in quadrants on pre-cultured Vero cells (Vero, CCL-81) in 24-well tissue culture plates. Plates were examined for 7 days following virus injection using an inverted microscope (Hund-Germany) to identify cytopathic effects (CPE). The infectious virus was identified by measuring the 50% tissue culture infectious dose (TCID 50) employing the Reed and Muench equation^38^.

### Cell culture and human rotavirus infectivity titer estimation

Caco-2 cells provided by the International Center for Advanced Research (ICTAR—Egypt) were grown in Minimum Essential Medium containing 10% fetal bovine serum, 100 U/mL penicillin, and 100 U/mL streptomycin at 37 °C in a humidified 5% CO_2_ incubator until a confluent sheet of cells was obtained. Human rotavirus was first activated for 30 min by treatment with 10 µg/mL trypsin at 37 °C, then propagated on the Caco-2 cell line in a 24-well plate at 37 °C in a humidified 5% CO_2_ incubator. The plate was checked daily for CPE. The infectious virus was enumerated by determining the 50% tissue culture infectious dose (TCID 50) using the Reed & Muench equation^38^. The virus stock was stored at -80 °C until use.

### Cytotoxicity assay

The cytotoxicity of the green SeNPs produced by the *C. roseus* extract on Caco2 cells and Vero cells were assessed. Cells were seeded on 96-well plates until a confluent layer was obtained. Then, cells were incubated with different concentrations of SeNPs, and the morphology of the cells was investigated daily for up to 72 h by inverted light microscopy according to Wu et al.^39^, for any morphological alteration.

### In vitro assays for the antiviral effect of the green SeNPs on human rotavirus and adenovirus type 40

For human rotavirus, the infectivity of rotavirus was activated by adding 10 µg/mL trypsin for 30 min. at 37 °C. A 100 µL aliquot of the activated rotavirus at a final concentration of 1 × 106 TCID50/ml was mixed with an equal volume of nanoparticles (5 µg/mL to 10 mg/mL) at the prepared concentrations. The same treatment was performed for human adenovirus. The mixture of different concentrations of nanoparticles containing the viruses was incubated for 1 h. After CaCo2 cells and Vero cells adhered onto a 96-well plate, the cells were incubated with the previous concentrations of nanoparticles (5 µg/mL to 10 mg/mL) containing human rotavirus and adenovirus for 7 days at 37 °C in a CO_2_ incubator under 5% CO_2_. As a positive control, cells inoculated with the viruses only, without nanoparticles, were also included in the assay. During the incubation period, cells were checked daily under the inverted microscope for CPE. The inhibitory effect of the different metal nanoparticles on virus infectivity was measured by determining the 50% tissue culture infectious dose (TCID 50) using the Reed and Muench equation^38^.

### Assessment of anticancer activity against hepatocarcinoma cells

The potential anticancer activity of SeNPs biosynthesized from *C. roseus* extract, at a concentration of 5.385 mg/mL, was investigated in vitro using normal liver epithelial cells (THLE-2) and the HepG2 to evaluate cytotoxicity through the MTT assay. Test samples were used at 200 mg/mL and serially diluted twice on precultured cell lines’ well plate for 24 h of treatment at 37 °C after decanting the growth medium. The treated cell lines were examined under a microscope to detect changes in morphology and detached cells. Dead cells were washed out with phosphate buffer saline (PBS-0.05% Tween) at pH 7.2 ± 0.2. Remaining live cells had been treated with 0.5% 3-(4,5-dimethylthiazol-2-yl)-2,5-diphenyltetrazolium bromide (MTT) stain at 25 µl per well. Plates were incubated for 3–4 h at 37 °C. After the incubation period, each well received 0.05 mL of DMSO for 30 min on a plate shaker to dissolve the formazan crystals, which are insoluble in aqueous solutions. After shaking the plates for 30 min, each well’s color absorbance was measured at 570 nm^40^ (using an enzyme-linked immunosorbent assay (ELISA) reader (Biotek 800, USA) to calculate the cell viability percent by comparing the treated and untreated cells’ absorbances as follows: Cell viability percentage = (OD of treated cells / OD of untreated cells) multiplied by 100^41^. The IC50 of SeNPs was estimated using the Master-plex-2010 program. This experiment was repeated at least 3 times^42^.

### Statistical analysis

All experiments were performed in triplicate (*n* = 3), and the data are presented as mean ± standard deviation (SD). Statistical analysis was performed using IBM SPSS Statistics version 16. Differences among groups were analyzed using one-way analysis of variance (ANOVA) followed by Tukey’s post-hoc multiple comparison test. Statistical significance was considered at *p* < 0.05.

### Docking analysis

The selected compounds and their interaction with SeNPs were molecularly docked using the MOE program. The Discovery Studio Client (version 4.2) was utilized to locate it in^20,21^. The Confirmation Examination module of AutoDock Vina was utilized to reduce the energy of the acquired conformations after conducting a thorough conformational analysis to an RMS gradient of 0.01, and a Molecular dynamics simulation of these metal complexes was made through GROMACS^22^ in water solvent with AMBER/CHARMM with metal-specific parameters at 300 K. Docking with the twinned 3.35 A structure of *S. aureus* Gyrase complex with Ciprofloxacin and DNA, PDBID:2XCT^23^ and *Salmonella typhi* OmpF complex with Ciprofloxacin PDBID:4KRA^24^, PCSK9:EGFA (pH 7.4)(PDBID3GCX)^25^, structural analysis of KSHV thymidylate synthase(PDBID:5H38)^26^, x-ray structure of a pokeweed antiviral protein, coded by a new genomic clone, at 0.23 nm resolution. A model structure provides a suitable electrostatic field for substrate binding.(PDBID:1PAP)^27^, and high-resolution structure of recombinant dianthin antiviral protein-potent anti-HIV agent (PDBID:1LP8)^28^. Ten distributed docking simulations were run with the default parameters. Conformations were made based on the overall data organization, the E conformation, and the right placement of relevant amino acids in the binding pocket of each protein^29^.

#### Characterization of the biosynthesized SeNPs

The green SeNPs produced by the *C. roseus* extract have been investigated employing a variety of physicochemical approaches, which included ultraviolet-visible (UV-Vis), high-resolution transmission electron microscopy (HRTEM), zeta potential, scanning electron microscopy (SEM) with energy dispersive X-ray (EDAX), X-ray diffraction (XRD), and Fourier transform infrared (FTIR).

## Results and discussion

SeNPs possess distinctive physicochemical properties that support their broad applications in medicine, microbiology, and environmental science^7^. However, the production efficiency of biologically synthesized SeNPs remains an important consideration for their broader application^7^. Plant-mediated green synthesis using *Catharanthus roseus* has attracted considerable interest due to the plant’s rich phytochemical composition and reported pharmacological activities, including antibacterial, antiviral, antifungal, and anticancer properties^43,44^. In the present study, SeNPs were synthesized using *C. roseus* extract and subsequently characterized to evaluate their physicochemical features and biological activities. It should be noted that SeNPs were prepared in a single synthesis batch and used for all subsequent experiments; therefore, batch-to-batch reproducibility was not assessed in this study. Future investigations should focus on evaluating synthesis reproducibility and scalability to facilitate potential large-scale production.

### LC–MS profiling of C. roseus extract

**Fig. 1:**
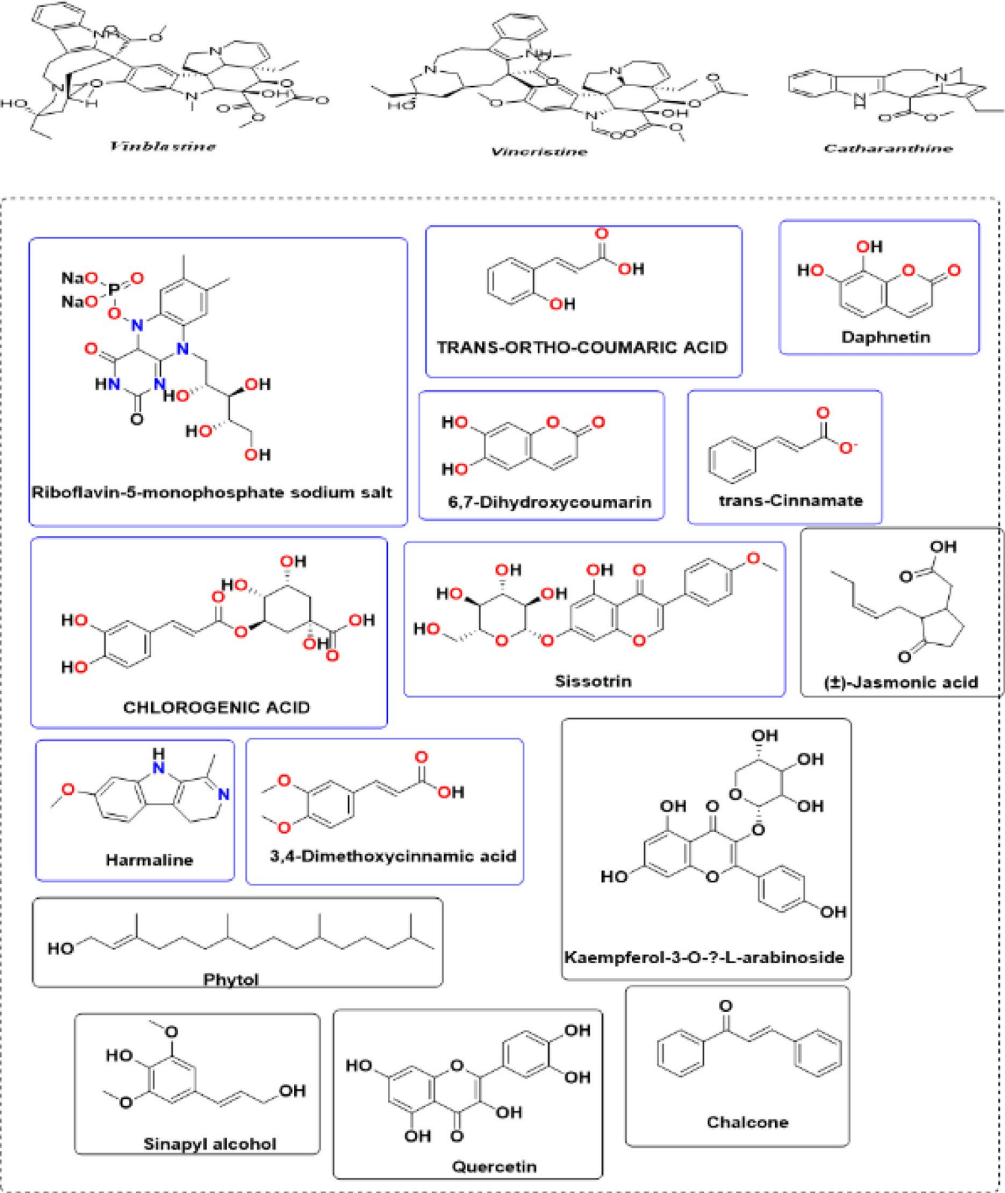
Chemical composition of the alkaloids and phenolic compounds utilized LC-MS.

Unlike many previously reported plant-mediated selenium nanoparticle (SeNP) synthesis studies that rely on crude extracts without detailed metabolite characterization, the present work integrates metabolomic profiling with nanoparticle synthesis to elucidate the potential role of specific phytochemicals in the reduction and stabilization processes^13^. The phytochemical composition of the *Catharanthus roseus* extract was therefore investigated using liquid chromatography–mass spectrometry (LC–MS) to identify the secondary metabolites potentially involved in nanoparticle biosynthesis^45^. Although LC–MS/MS identified several redox-active phytochemicals in the extract, their direct role in nanoparticle reduction and stabilization was not experimentally verified and is proposed based on spectroscopic evidence and previous literature. The LC–MS chromatographic profile obtained in positive ionization mode revealed a chemically complex extract containing phenolic acids, flavonoids, coumarins, terpenoids, and alkaloid derivatives (Fig. 1 and Table S1). These classes of compounds are widely recognized for their antioxidant and redox properties and are frequently implicated in the green synthesis of metallic nanoparticles^46,10^. Among the detected metabolites, riboflavin-5′-monophosphate sodium salt hydrate (m/z 457.1541; peak area 1.77 × 10^9^) was identified as the predominant compound. This flavin-derived molecule exhibits strong redox activity and can participate in electron-transfer reactions that facilitate the formation of nanoparticles. Chlorogenic acid (m/z 355.077; peak area 8.56 × 10^8^), a well-known phenolic antioxidant with strong radical-scavenging capacity, was also detected in high abundance. Additional phenolic acids identified in the extract included trans-ortho-coumaric acid, trans-cinnamate, and 3,4-dimethoxycinnamic acid, which are known contributors to the antioxidant potential of plant extracts. Several flavonoids were also identified, including quercetin, kaempferol-3-O-α-L-arabinoside, and sissotrin. These compounds are widely reported for their anti-inflammatory, antioxidant, and cytoprotective properties and are known to function as effective electron donors during metal ion reduction processes^46,47^. The detection of chalcone, an important precursor in flavonoid biosynthesis, further indicates an active flavonoid metabolic pathway in *C. roseus*. Additionally, two coumarin derivatives—6,7-dihydroxycoumarin (daphnetin) and related compounds—were detected, which may contribute to the antioxidant capacity and photoprotective properties of the extract. The β-carboline alkaloid harmaline (peak area ≈ 2.6 × 10^8^) was identified as a major alkaloidal component and has been reported to exhibit antimicrobial and neuroprotective activities. Other notable metabolites included phytol, a diterpenoid alcohol; sinapyl alcohol, a phenylpropanoid involved in lignin biosynthesis; and (±)-jasmonic acid, a lipid-derived oxylipin associated with plant defense signaling. The presence of these metabolites suggests a strong connection between phenolic metabolism and plant defense responses. Overall, seventeen metabolites were identified in the extract, with phenolic compounds representing approximately 70% of the detected constituents, followed by alkaloids (≈ 12%). The abundance of redox-active phytochemicals highlights the strong antioxidant capacity of *C. roseus* extract and supports its potential role as a natural reducing and stabilizing system for the biosynthesis of selenium nanoparticles.

### Role of phytochemicals in selenium nanoparticle biosynthesis

The phytochemicals identified in the *C. roseus* extract likely play a central role in the biosynthesis of selenium nanoparticles by acting as natural reducing, capping, and stabilizing agents. Phenolic compounds are particularly important in this process because of their ability to donate electrons and participate in redox reactions, enabling the reduction of metal ions during green nanoparticle synthesis^8^. The biosynthesis of SeNPs generally occurs through three sequential stages: reduction, nucleation, and stabilization. In the reduction stage, selenium ions (Se⁴⁺) derived from sodium selenite (Na_2_SeO_3_) are reduced to elemental selenium (Se⁰) through electron donation from phenolic hydroxyl groups (–OH). During this process, phenolic compounds are oxidized to quinone or carbonyl intermediates while generating elemental selenium atoms. These atoms subsequently aggregate to form nanoscale selenium nuclei that initiate nanoparticle formation. In the subsequent capping stage, oxygen-containing functional groups such as hydroxyl, carbonyl, carboxylate, and phosphate groups interact with the nanoparticle surface through Se–O interactions, creating an organic protective layer. This coating prevents uncontrolled aggregation and regulates nanoparticle size and surface charge. Finally, stabilization occurs through hydrogen bonding and π–π interactions between phytochemical ligands and the nanoparticle surface, forming a stable colloidal network that enhances nanoparticle dispersibility and biocompatibility. As a result, the biosynthesized SeNPs exhibit characteristic orange-red coloration and improved antioxidant and antimicrobial properties due to the phenolic functionalization of their surface^48,49^. These findings indicate that the phytochemicals present in *C. roseus* extracts do not merely function as reducing agents. Instead, they form an integrated biochemical system that regulates multiple stages of SeNP formation, including electron transfer, surface passivation, and nanoparticle stabilization. This coordinated mechanism ultimately influences the morphology, physicochemical properties, and biological performance of the synthesized nanoparticles.

### Phytochemical–protein interaction network using SeNP biosynthesis

**Fig. 2:**
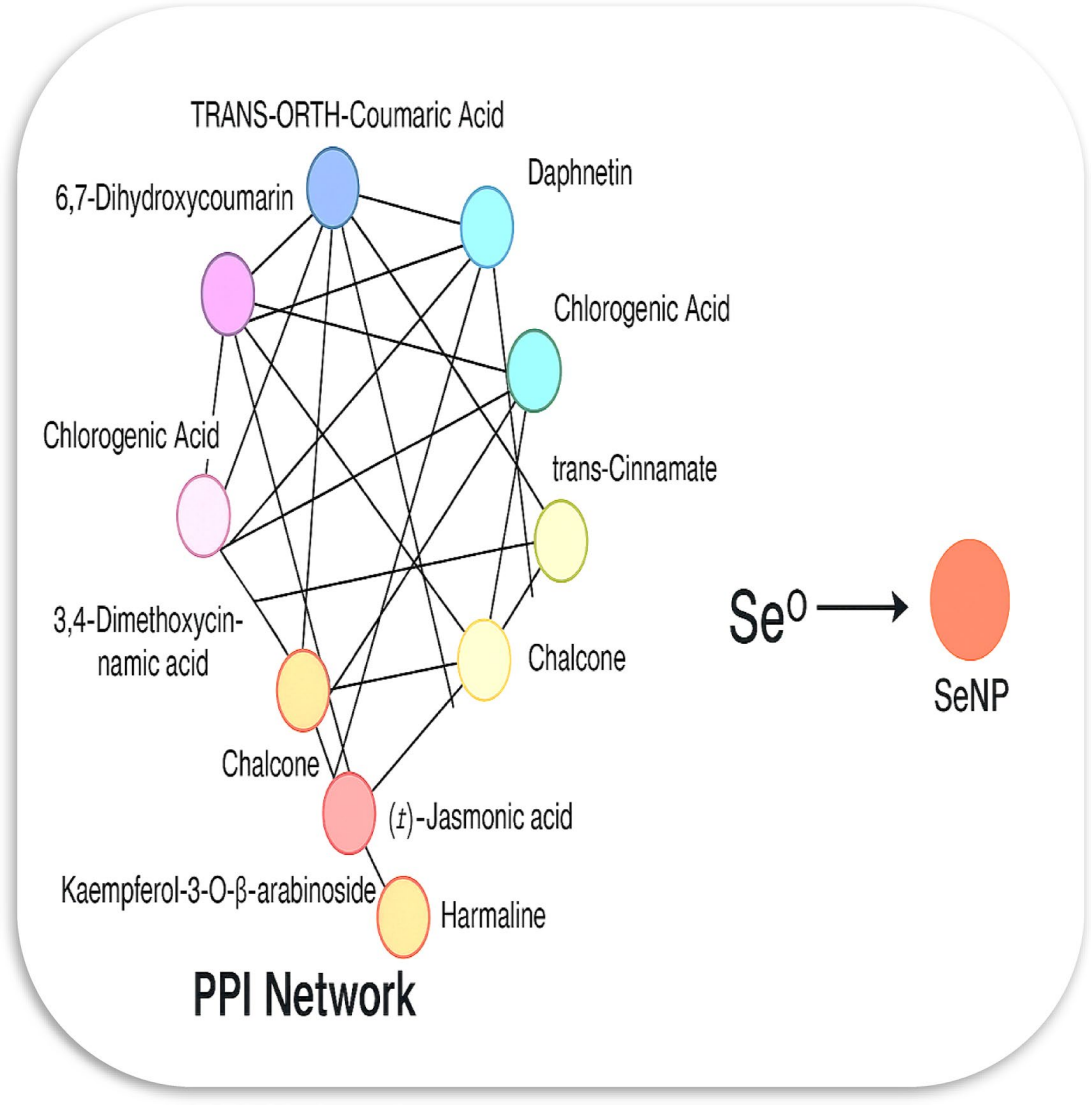
The network illustrates a protein-protein interaction (PPI)like network of key phytochemicals identified from LC-MS analysis. Each node symbolizes an active compound, while connecting lines indicate potential redox or synergistic interactions that could enhance electron flow during bioreduction.

To further explore the mechanistic basis of selenium nanoparticle formation, a phytochemical–protein interaction network was constructed based on the metabolites identified through LC–MS analysis (Fig. 2; Table 1). This model integrates 15 bioactive phytochemicals, 20 redox-active proteins, and three selenium species (selenite, Se^0^ nuclei, and SeNPs) to illustrate potential electron-transfer pathways involved in nanoparticle biosynthesis. Several phenolic and flavonoid compounds—including quercetin, kaempferol-3-O-α-L-arabinoside, chlorogenic acid, and coumarin derivatives—are predicted to activate the NFE2L2 (NRF2) signaling pathway via inhibition of KEAP1. Activation of this pathway may stimulate the expression of detoxifying and antioxidant enzymes such as NQO1, HMOX1, TXNRD1, and GPX1, which play important roles in cellular redox homeostasis. These enzymes are capable of mediating thiol- and flavin-dependent redox reactions that facilitate the conversion of selenite (SeO_3_^2−^) into elemental selenium (Se⁰). Riboflavin-5′-monophosphate may serve as a key flavoprotein cofactor linking electron-transfer enzymes such as NQO1, ferredoxin-NADP⁺ reductase (FNR), and FDX1. Additional enzymes, including thioredoxin reductase (TXNRD1) and glutathione reductase (GSR), contribute to the regeneration of cellular reducing equivalents (NADPH → GSH/TXN), thereby maintaining the redox conditions necessary for selenium reduction. These processes ultimately promote the nucleation of elemental selenium and the formation of stable selenium nanoparticles. Overall, the proposed network supports a bio-inspired electron-relay mechanism in which phytochemical antioxidants interact synergistically with endogenous redox systems to drive the biosynthesis of selenium nanoparticles. By integrating plant metabolomics with nanobiotechnology, this approach provides deeper mechanistic insight into green nanoparticle synthesis and highlights a sustainable strategy for the development of functional selenium nanostructures. It should be noted that the proposed phytochemical–protein interaction network represents a predictive and integrative model derived from metabolomic identification and computational analysis. While this framework provides valuable insight into potential electron-transfer pathways involved in SeNP biosynthesis, it does not constitute direct mechanistic proof, and experimental validation will be required in future studies to confirm these interactions. Similar integrative computational approaches have been used to explore nanoparticle–biomolecule interactions, although their interpretations should be considered supportive rather than definitive^50,51^.

**Table 1:** Key phytochemical protein Interactions driving SeNP formation.

Compound	Primary protein target	Mechanistic role	Expected effect on Se reduction
Riboflavin-5′-phosphate	NQO1 / FNR / FDX1	Electron mediator (flavin cofactor)	Enhances electron transfer to SeO_3_^2−^
Quercetin	KEAP1 / NRF2	Activates antioxidant pathway	Increases NQO1/TXNRD1 levels
Kaempferol-3-O-α-L-arabinoside	NRF2 / GPX1	Redox modulator	Boosts glutathione cycle
Chlorogenic acid	NQO1 / TXN	ROS scavenging / NADPH transfer	Promotes Se^0^ nucleation
Coumarins (6,7-Dihydroxycoumarin, Daphnetin)	NQO1 / PRDX1	Quinone-type redox cycling	Stimulates selenite reduction
Sinapyl alcohol	GPX1 / GSR	GSH cycle component	Facilitates GSH-dependent Se reduction
(±)-Jasmonic acid	NRF2	Stress signaling	Indirect induction of antioxidant proteins
Harmaline	MAOA	β-Carboline alkaloid; off-pathway regulator	Minor ROS modulation

### Characterization of the green SeNPs

Green SeNPs manufactured by *C. roseu*s extract were investigated using UV-Vis, HRTEM, zeta potential, SEM, XRD, and FTIR techniques, as illustrated below.

### Biosynthesized SeNPs using UV-Vis spectroscopy

**Fig. 3:**
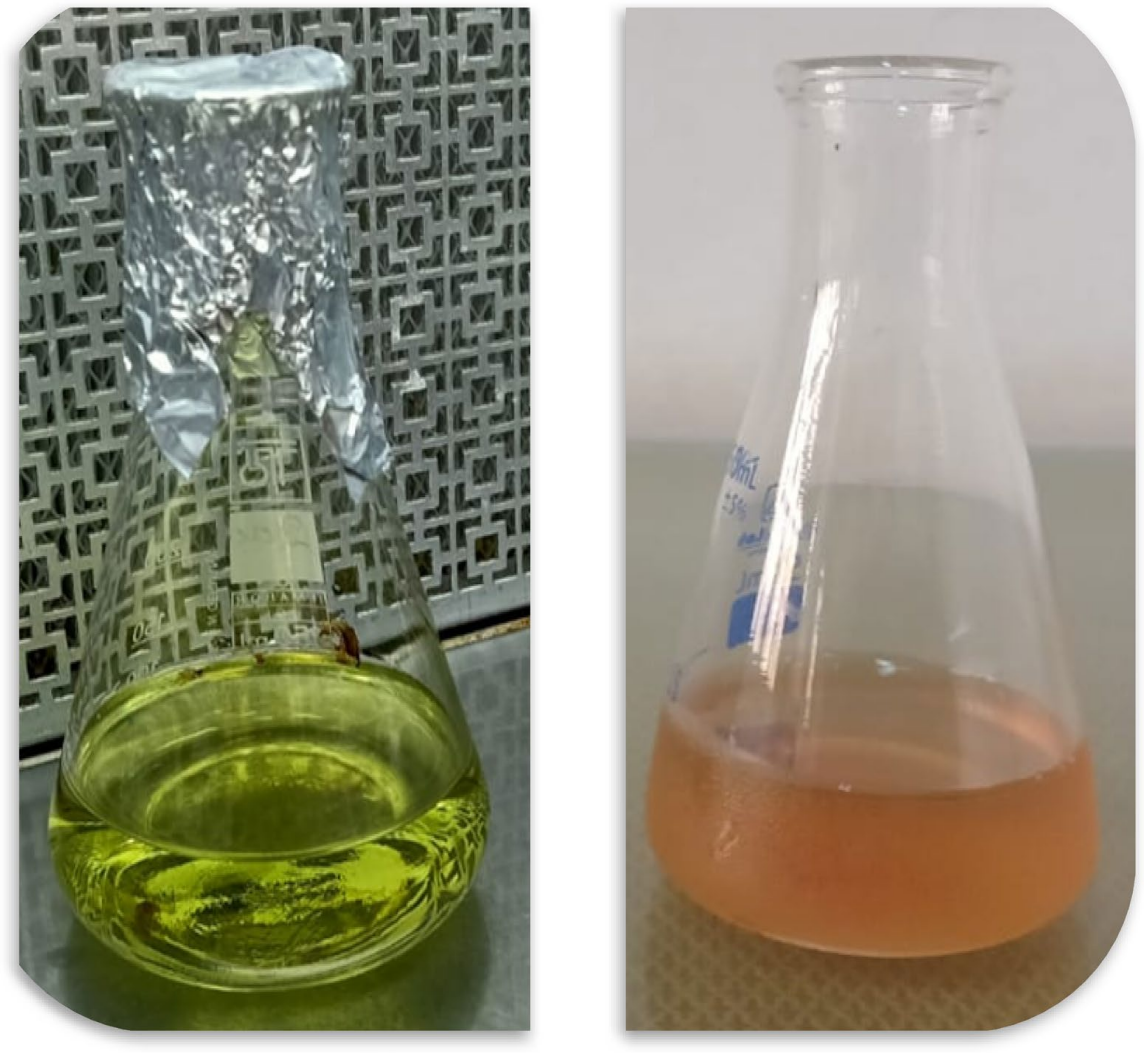
Visual observation of the SeNPs biosynthesized by the *C. roseus* extract.

**Fig. 4:**
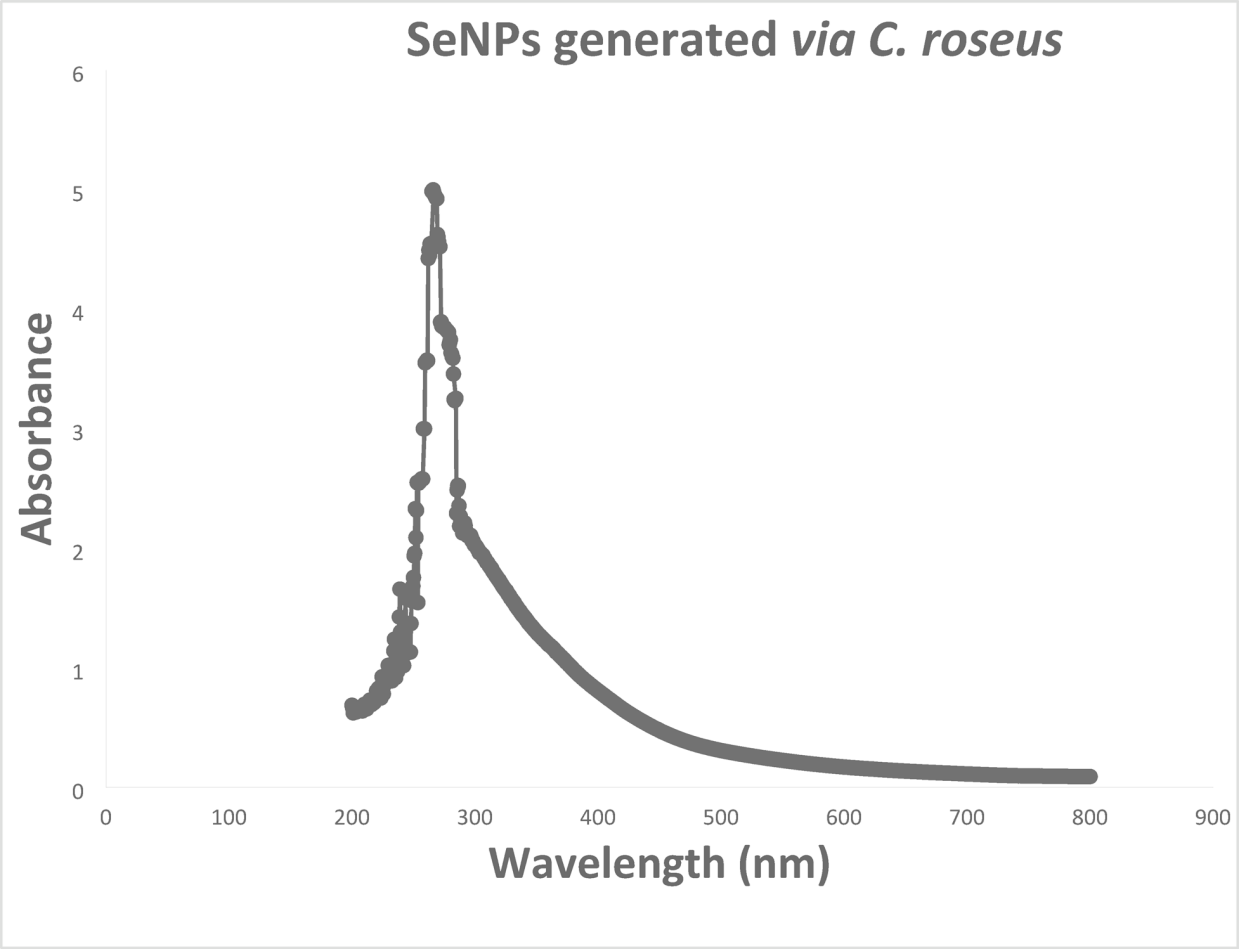
UV-visible absorption spectra of SeNPs generated *via* the *C. roseus* extract.

Visual observation indicated that the reaction mixture of SeNPs synthesized with *C. roseus* extract changed from a pale green to a pinkish-red hue (Fig. 3), reflecting the formation of nanoparticles. This color transition is likely influenced by variations in particle size and surface plasmon resonance (SPR). While this provides a convenient qualitative indicator of synthesis, no time-resolved UV-Vis measurements were conducted to monitor the progression of the characteristic SeNP absorption peak. Implementing such kinetic studies in future work would allow quantitative tracking of nucleation and growth dynamics, providing deeper insight into particle formation, size evolution, and stabilization mechanisms. SeNPs typically exhibit distinct spectral characteristics owing to their distinctive optical resonance, known as SPR, which occurs as a result of NP shape and size^52^. In the present investigation, UV-vis spectroscopy findings from *C. roseus* reveal that it can form SeNPs extracellularly by reducing Se^+4^. Figure 4 demonstrates a UV-visible spectroscopy analysis of SeNP generation using *C. roseus* extract, exhibiting a distinct and typical SPR band at 266 nm. The UV peak position and sharpness indicate the NPs are small, well-dispersed, and feature adequate crystallinity^53^. Similar to this, Fouda et al.^54^ reported an SPR wavelength band at 266 nm in UV-visible spectra of synthesized SeNPs generated through *Portulaca oleracea*. On the other hand, the spectrum of the SPR for various other plant-biosynthesized SeNPs varied between 210 and 280 nm^55–57^.

### HRTEM

**Fig. 5:**
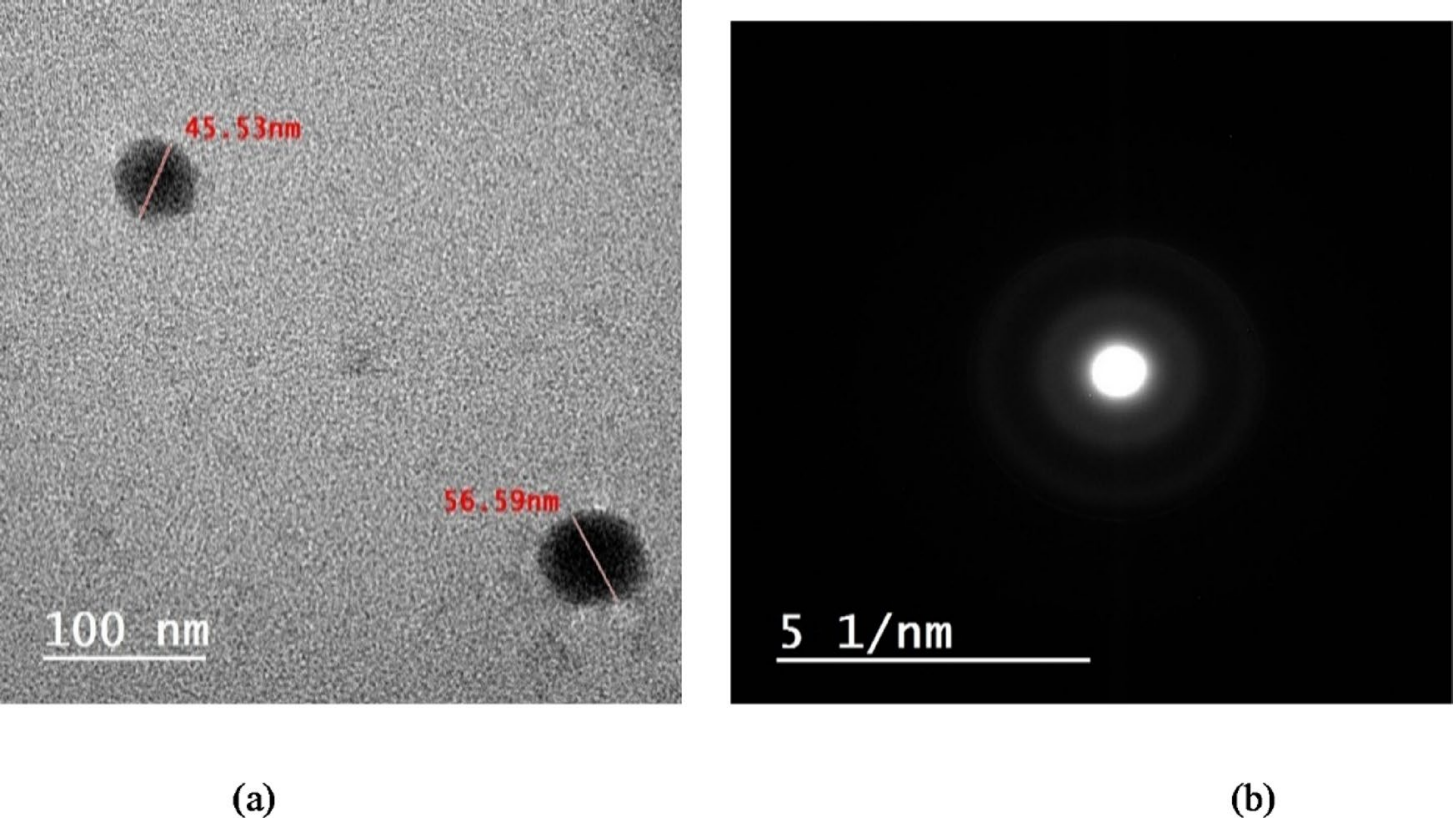
Shows (**a**) TEM micrographs of green SeNPs synthesized using *C. roseus* extract and (**b**) the SAED pattern of these SeNPs.

The morphology, size, and crystallinity of the biosynthesized SeNPs were examined using transmission electron microscopy (TEM) (Fig. 5). The TEM micrographs reveal predominantly spherical to quasi-spherical nanoparticles with minimal aggregation, confirming successful nanoscale formation. Scale bars are provided for accurate dimensional reference. Particle size distribution analysis, performed by measuring the diameter of more than 150 individual nanoparticles using ImageJ software, showed a size range of 8.6–65.61 nm with an average core diameter of 32.5 ± 12.1 nm. This core size represents the true inorganic selenium nuclei and is typically smaller than hydrodynamic values reported by DLS in the literature, since TEM does not capture the surrounding solvation layer and phytochemical capping shell. The corresponding selected-area electron diffraction (SAED) pattern exhibits well-defined concentric diffraction rings that index to multiple lattice planes, confirming a polycrystalline nature of the SeNPs, which is consistent with previously reported biogenic nanoparticles^58,59^. Collectively, these microstructural features and the narrow size distribution support the conclusion that phytochemicals in the *C. roseus* extract facilitate efficient reduction, capping, and structural stabilization, resulting in uniform, crystalline SeNPs. The larger hydrodynamic diameter obtained by DLS compared with the TEM core size can be attributed to the presence of a hydration shell and phytochemical capping layer surrounding the SeNPs in aqueous suspension.

### Zeta analysis

Estimating zeta potential and evaluating particle size distribution are crucial for understanding nanoparticle interactions and suspension stability^60^. In the present study, the zeta potential, hydrodynamic diameter, and polydispersity index (PdI) of the biosynthesized SeNPs were determined, as shown in Table 2. The recorded zeta potential, size distribution number, and PdI for SeNPs synthesized using *C. roseus* extract were − 3.55 mV, 232.9 d.nm, and 0.499, respectively. Zeta potential and hydrodynamic size were measured directly using the as-prepared aqueous SeNP suspension without further dilution, and measurements were conducted under the default conductivity settings of the instrument.Although the zeta potential value falls below the threshold typically associated with strong electrostatic stabilization (> |30| mV), the observed negative charge suggests the presence of weak electrostatic repulsion, which can still contribute to reduced nanoparticle aggregation^61^. This charge is likely attributed to phytochemical constituents present in the plant extract, acting as natural capping and stabilizing agents, thereby supporting colloidal stability. In addition, the obtained PdI value of 0.499 indicates a moderately polydisperse size distribution. While synthetic nanoparticles commonly exhibit PdI values < 0.3, such narrow distributions are less typical for green-synthesized nanomaterials. Plant-derived SeNPs frequently display PdI values in the range of 0.4–0.6 due to the heterogeneous nature of the phytochemical reducing and capping agents. Importantly, this level of polydispersity does not impair their functional performance, as the organic corona surrounding biogenic SeNPs often enhances colloidal stability and biological activity. The observed negative surface charge, combined with the functional phytochemical coating, therefore supports the suitability of the synthesized SeNPs for biological applications despite their moderate polydispersity.

**Table 2:** The SeNPs formed *via* the *C. roseus* extract, zeta potential, the size distribution by number, and PdI.

Sample	Zeta potential (mV)	Size distribution number (d.nm)	PdI
SeNPs from *C. roseus* extract	-3.55 ± 4.5	232.9 ± 73.80	0.499 ± 0

#### SEM-EDAX of the biosynthesized SeNPs

**Fig. 6:**
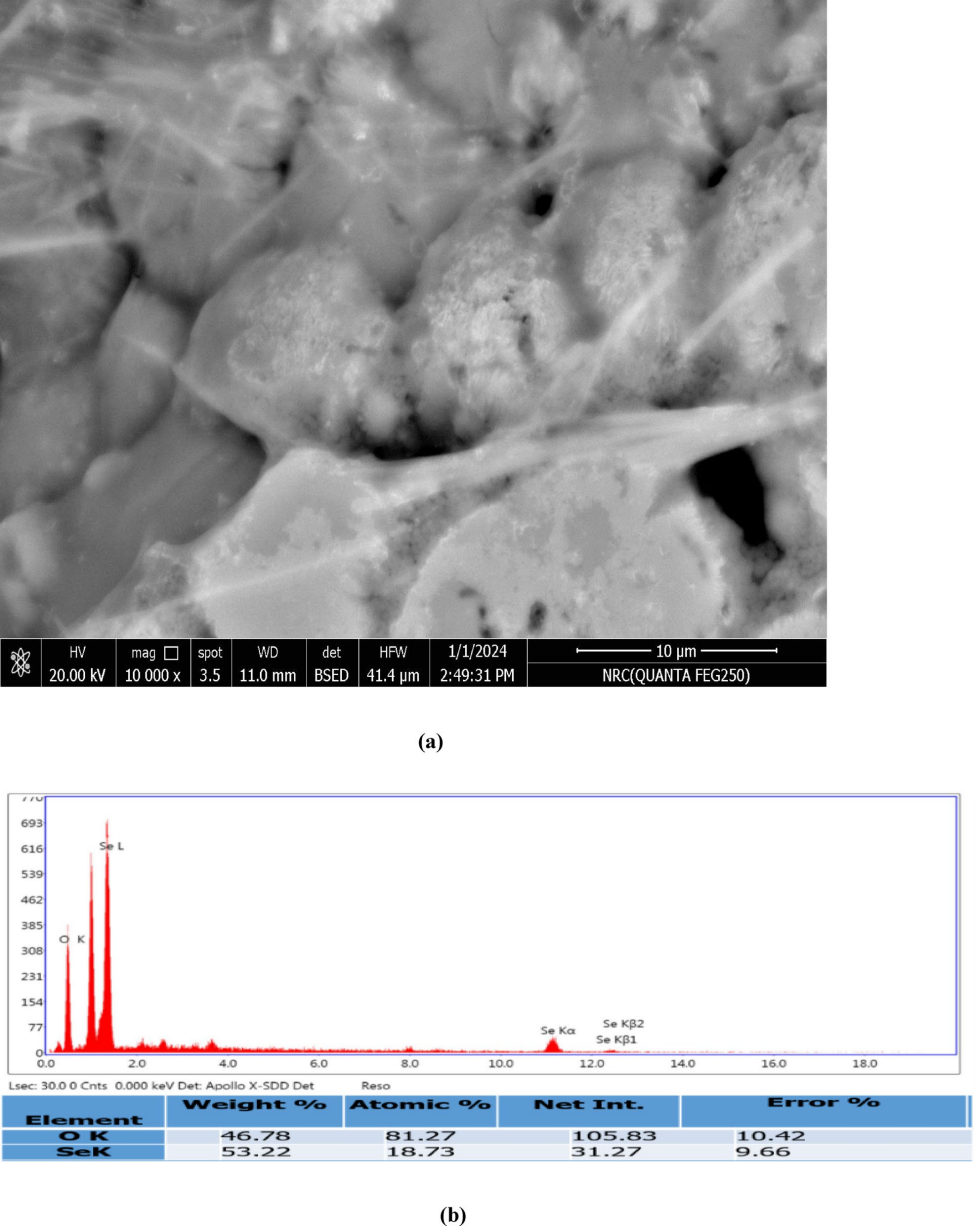
SeNPs generated from the *C. roseus* (**a**) SEM and (**b**) EDX.

The accompanying EDX spectrum (Fig. 6b) confirms the elemental composition of the produced nanoparticles, with selenium as the dominant signal. The detected weight percentages of Se (53.22%) and O (46.78%) correspond to atomic percentages of 18.73% and 81.27%, respectively. This significant oxygen signal is attributed to the expected formation of a thin native selenium oxide (SeO_2_) layer on the nanoparticle surface, combined with surface-adsorbed oxygen-containing phytoconstituents from the biological extract (e.g., hydroxyl groups, residual water). This surface oxidation is a common characteristic of biogenic SeNPs and does not indicate bulk impurity; rather, it contributes to the particles’ colloidal stability and surface reactivity, which are relevant for their intended application^62,63^. Thus, the EDX data confirms the successful synthesis of SeNPs with a characteristic surface composition. The combined evidence from SEM morphology, statistically validated size distribution, and elemental analysis confirms the controlled structural formation of stable SeNPs.”

### X-ray diffraction

**Fig. 7:**
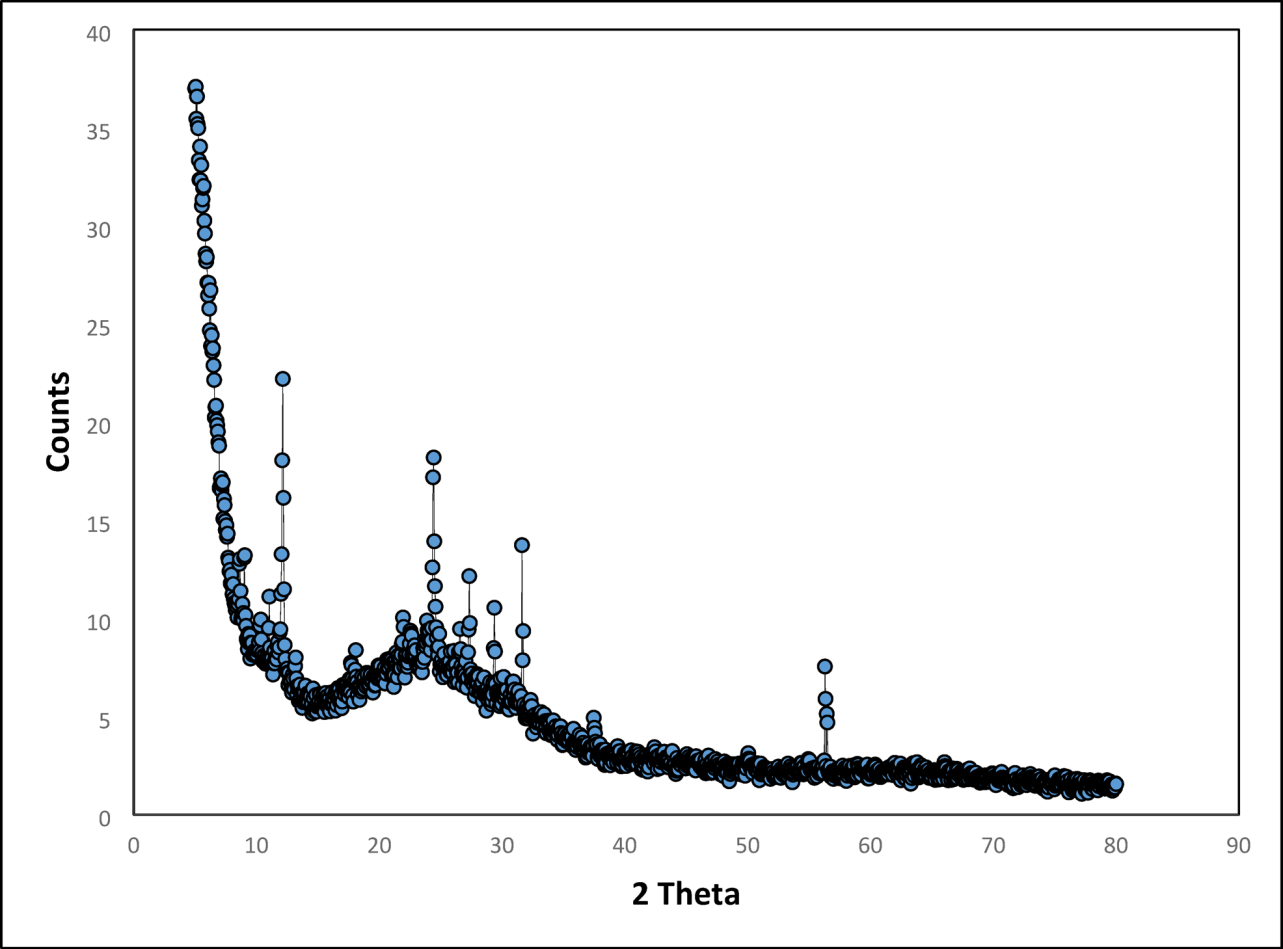
X-ray diffraction pattern of green SeNPs produced by *C. roseus* extract.

The crystalline nature and phase composition of the SeNPs were examined using X-ray diffraction (XRD)^58,64^. As shown in Fig. 7, the diffraction pattern displays the characteristic reflections of trigonal (hexagonal) selenium, with prominent peaks at 2θ = 23.90°, 29.37°, and 31.65°, corresponding to the (100), (101), and (111) planes, respectively (JCPDS #06-0362)^65^. These peaks confirm the successful bio reduction of selenite to crystalline Se⁰. The noticeable broadening of these reflections, consistent with the Debye–Scherrer relationship, indicates nanoscale crystallite dimensions. In addition to the selenium peaks, several low-angle reflections were observed (e.g., 11.04°, 12.14°, and 21.92°). These do not match any known allotropic form of elemental selenium and are instead attributed to semi-crystalline organic residues from the *C. roseus* extract. The sharp diffraction peaks, therefore, represent the crystalline Se core, whereas the broad low-intensity features arise from the amorphous or semi-crystalline phytochemical capping layer that coats the nanoparticle surface. Such signals are commonly reported in biogenic nanomaterials, where phytoconstituents—particularly phenolics and alkaloids—participate in capping and stabilization processes^18^. Their presence in the XRD pattern supports the formation of an organic-rich surface layer, in agreement with the strong oxygen signal observed in the EDX analysis (Fig. 6b). TEM analysis further confirms the nanoscale size and morphology of the SeNPs, showing spherical to slightly polygonal particles consistent with the crystalline core sizes derived from XRD. Comparison with previous reports indicates that these morphological and structural features are typical of plant-mediated SeNPs^58,64^. Taken together, the XRD and TEM data, in the context of prior literature, provide a comprehensive understanding of the SeNPs as crystalline cores enveloped by an amorphous or semi-crystalline phytochemical matrix—a structure widely associated with enhanced colloidal stability and improved functional performance in biological and catalytic applications^66^.

### Fourier transform infrared spectroscopy

**Fig. 8:**
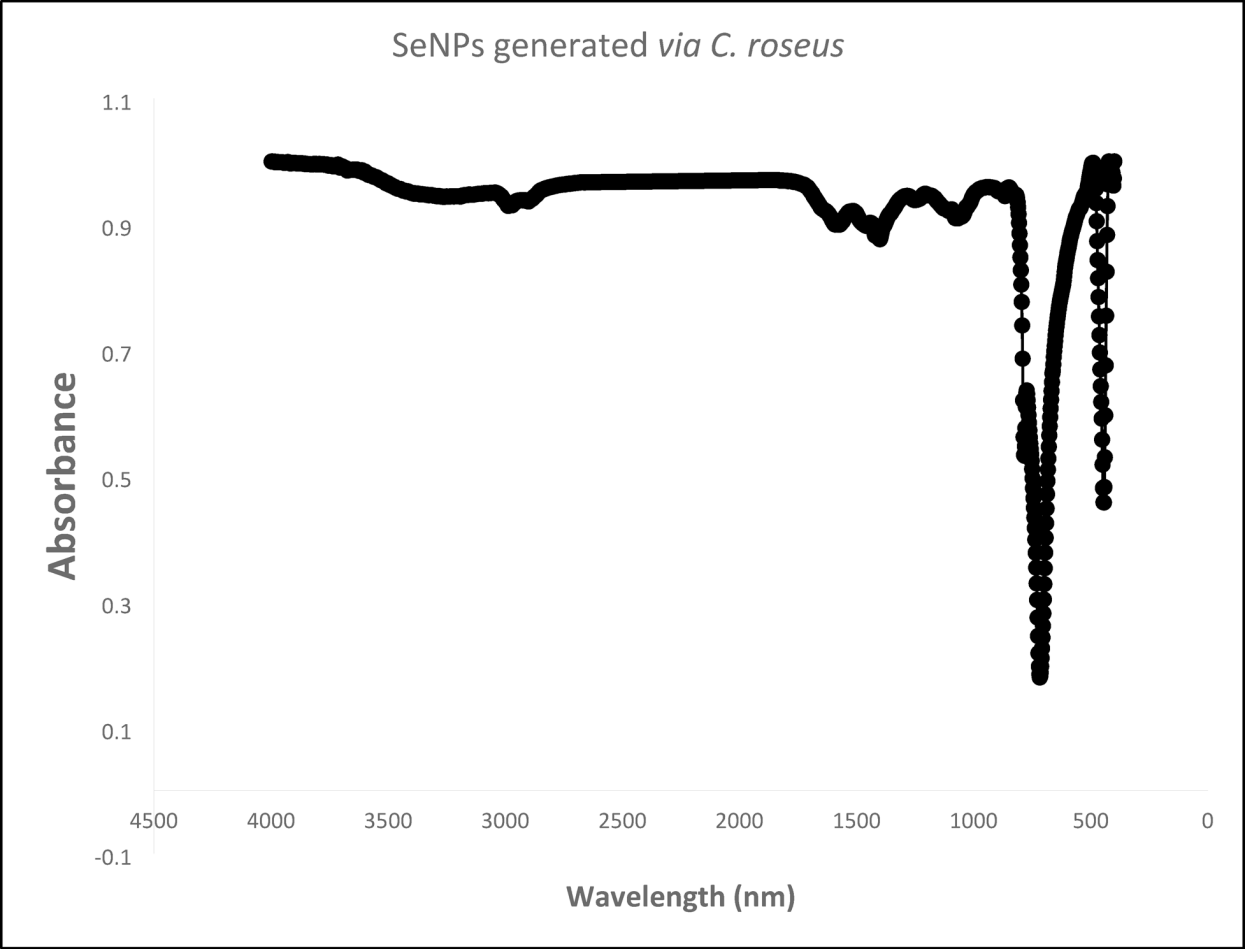
FTIR spectrum of SeNPs generated *via* the *C. roseus* extract.

The FTIR spectrum of the *C. roseus*-synthesized SeNPs (Fig. 8) exhibited distinct vibrational peaks. Analysis of these peaks, which correlate directly with the major phytoconstituents of *C. roseus*, provides mechanistic insight into the bioreduction and stabilization processes. FTIR analyses previously reported for *C. roseus* extracts commonly detect amines, amides, alkaloids, phenolics, ethers, and glycosides^58^. The band at ~ 1574 cm^−1^ is consistent with amide II vibrations (N–H bending / C–N stretching) plus aromatic C = C modes, which aligns with the presence of alkaloid N–H (e.g., from indole-alkaloids such as vindoline, catharanthine) or peptide/amide–like components rather than a classical carbonyl (C = O) stretch^67^. The prominent C–O stretch at 1066.77 cm^−1^ aligns with the ether and hydroxyl groups abundant in iridoid glycosides (e.g., loganin) and flavonoid sugar moieties present in the extract^68^. Furthermore, aromatic C–H bending vibrations at 783.90 and 717.01 cm^−1^ confirm the presence of aromatic systems from indole alkaloids and phenolic compounds^69^. The C–H stretches observed (~ 2987 cm^−1^) are consistent with the aliphatic chains of these terpenoid and alkaloid backbones^70^. Collectively, this spectroscopic evidence suggests that the hydroxyl groups of phenolic and terpenoid components likely act as the primary reductants, while the surface-active alkaloids and glycosides facilitate capping and stabilization via their amine and ether/hydroxyl groups, respectively. Finally, the characteristic vibrations at 444.51 and 418.38 cm^−1^ confirm the formation of pure SeNPs^71^. These biochemical interactions provide a plausible mechanism whereby phytoconstituents not only initiate reduction of selenium ions but also remain adsorbed as surface-passivating ligands, ensuring improved stability and purity of the biosynthesized SeNPs.

### Molecular docking investigation

**Fig. 9:**
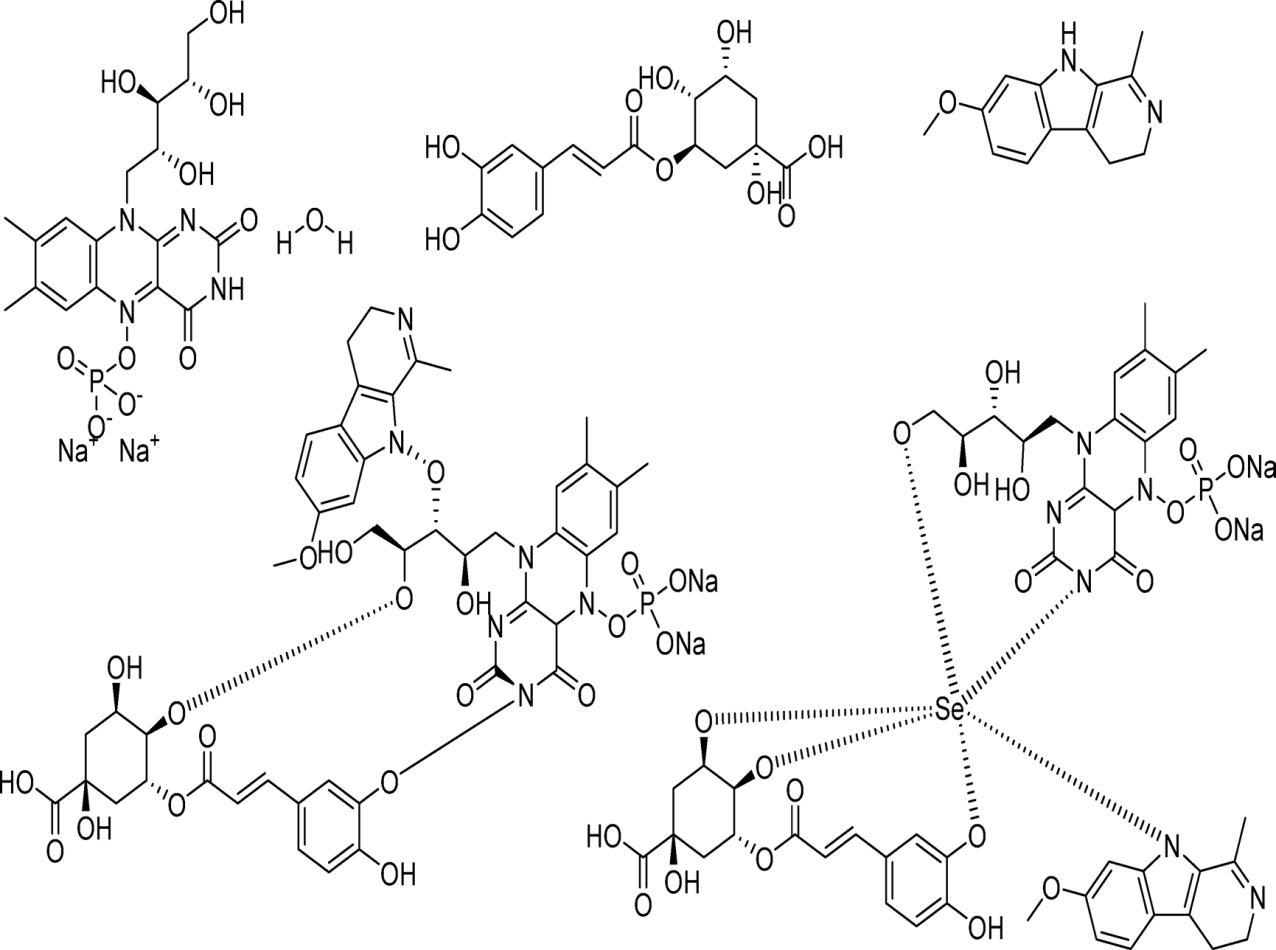
Selected compounds and their interaction with Se.

So we selected some of these compounds and their interaction to understand the interaction between the protein and the chelation of Se to convert to Se nanoparticles^72^, such as the following structures as displayed in Fig. 9.

The docking analysis of these compounds and chelation with Se with the twinned 3.35 A structure of S. aureus Gyrase complex with Ciprofloxacin and DNA, PDBID:2XCT^73^ and Salmonella typhi OmpF complex with Ciprofloxacin PDBID:4KRA^24^,PCSK9:EGFA (pH 7.4)(PDBID3GCX^74^, Structural analysis of KSHV thymidylate synthase(PDBID:5H38)^26^, X-RAY STRUCTURE OF A POKEWEED ANTIVIRAL PROTEIN, CODED BY A NEW GENOMIC CLONE, AT 0.23 NM RESOLUTION. A MODEL STRUCTURE PROVIDES A SUITABLE ELECTROSTATIC FIELD FOR SUBSTRATE BINDING.(PDBID:1PAP)^75^, and HIGH RESOLUTION STRUCTURE OF RECOMBINANT DIANTHIN ANTIVIRAL PROTEIN-POTENT ANTI-HIV AGENT(PDBID:1LP8^28^ using the MOE program. The Discovery Studio Client (version 4.2) was utilized to locate it in^20,21^. The Confirmation Examination module of AutoDock Vina was utilized to reduce the energy of the acquired conformations after conducting a thorough conformational analysis to an RMS gradient of 0.01, and a Molecular dynamics simulation of these metal complexes was made through GROMACS^22^ in water solvent with AMBER/CHARMM with metal-specific parameters at 300 K.

### Antimicrobial activity of biosynthesized SeNPs

The antimicrobial activity of the biosynthesized SeNPs was evaluated by measuring inhibition zone diameters together with minimum inhibitory concentration (MIC) and minimum bactericidal concentration (MBC) values, providing a comprehensive assessment of both growth-inhibitory and bactericidal potential^76^. The results indicate that SeNPs exhibited greater antimicrobial activity than the crude plant extract, suggesting that nanoparticle formation enhanced the bioactivity of the phytochemicals involved in the green synthesis process. The largest inhibition zones were observed against the Gram-positive strains *Staphylococcus aureus* and *Bacillus subtilis* (32–35 mm), whereas moderate inhibition was detected for the Gram-negative bacteria *Escherichia coli* and *Pseudomonas aeruginosa* (16–25 mm). The antifungal pathogen *Candida albicans* also showed substantial susceptibility with an inhibition zone of approximately 30 mm (Table 3). Correspondingly, MIC values ranged from 10.5 to 105 µg/mL, while MBC values varied between 420 and 636.36 µg/mL (Table 4). The comparatively lower MIC values observed for Gram-positive bacteria indicate their higher susceptibility to SeNP treatment relative to Gram-negative species. This difference in susceptibility can largely be attributed to variations in cell envelope architecture and reactive oxygen species (ROS) generation. Gram-positive bacteria possess a thick but relatively permeable peptidoglycan layer that facilitates nanoparticle interaction and penetration, enabling rapid ROS accumulation at the cell surface^77^. Selenium nanoparticles are known to catalyze the formation of ROS species such as superoxide anions (O_2_•⁻), hydroxyl radicals (•OH), and hydrogen peroxide (H_2_O_2_), which induce oxidative stress leading to lipid peroxidation, protein denaturation, and DNA damage^78,79^. In contrast, Gram-negative bacteria contain an additional lipopolysaccharide-rich outer membrane that restricts nanoparticle penetration and reduces ROS interaction with the cell membrane, resulting in comparatively smaller inhibition zones and higher MIC values^77,80^. The antifungal activity observed against *C. albicans* may also be associated with ROS-mediated mechanisms. ROS generated by SeNPs can disrupt ergosterol-rich fungal membranes, impair mitochondrial activity, and induce apoptosis-like responses through oxidative stress pathways^81^. Additionally, phytochemical capping agents derived from *Catharanthus roseus* may further enhance antimicrobial effects due to the presence of redox-active functional groups that promote oxidative stress within microbial cells^82^. To place these findings in context, the antimicrobial performance of the synthesized SeNPs was compared with previously reported plant-mediated selenium nanoparticle systems (Table S2). Earlier studies commonly report inhibition zones in the range of 12–28 mm and MIC values between 25 and 200 µg/mL, depending on the plant extract and synthesis method used (Alizadeh et al., 2023; Ibrahim et al., 2024). The SeNPs synthesized in the present study showed comparable or slightly improved activity, particularly against Gram-positive bacteria, with inhibition zones up to 35 mm and MIC values as low as 10.5 µg/mL. However, it should be noted that standard antibiotics generally exhibit lower MIC values than nanoparticle systems, indicating that SeNPs should be considered supportive antimicrobial agents rather than direct replacements for conventional antibiotics. Overall, these findings suggest that the antimicrobial activity of the biosynthesized SeNPs arises from the combined influence of their nanoscale size, high surface reactivity, and the presence of bioactive phytochemical capping agents derived from *C. roseus*. While the observed activity is promising, further investigations are required to fully elucidate the mechanisms of action and to evaluate their potential synergistic effects with conventional antimicrobial drugs.

**Table 3:** Mean zones of growth inhibition of the biosynthesized SeNPs and reference drugs against test organisms.

Tested samples	Mean zone of growth inhibition (mm) ± STDEV					
	*B. subtilus*	*S. aureus*	*E. coli*	*P*. aeruginosa	*C. albicans*	*A. niger*
*C. roseus* extract	-ve	-ve	-ve	-ve	-ve	-ve
SeNPs from *C. roseus* extract	35 ± 1.73	32 ± 2.65	25 ± 3.46	16 ± 2	30 ± 2.65	-ve
Amp	13 ± 0.5	14 ± 1.32	18 ± 0.87	14 ± 1	n.d	n.d
Myco	n.d	n.d	n.d	n.d	15 ± 0.5	14 ± 0.5

Size of well 6 mm.

Zones of growth inhibition = diameter of well plus zone of growth inhibition; diameter of well = 6 mm. The mean zone of inhibition was determined from three independent results (=3; nd = not determined, STDEV=standard diffusion, Amp=Ampicillin, Myco=Mycostatin).

**Table 4:** Minimal inhibitory and bactericidal concentrations of biosynthesized SeNPs toward test microorganisms.

Test	*B. subtilus*	*S. aureus*	*E. coli*	*P*. aeruginosa	*C. albicans*
MIC Inhibition zone µg/mL	≤ 10.5	≤ 52.5	≤ 75	≤ 105	≤ 80
MBC Inhibition zone µg/mL	≥ 420	≥ 525	≥ 525	≥ 636.36	≥ 636.36

MIC: minimum inhibitory concentration, MBC: minimum bactericidal concentration.

### Antimicrobial docking

**Fig. 10:**
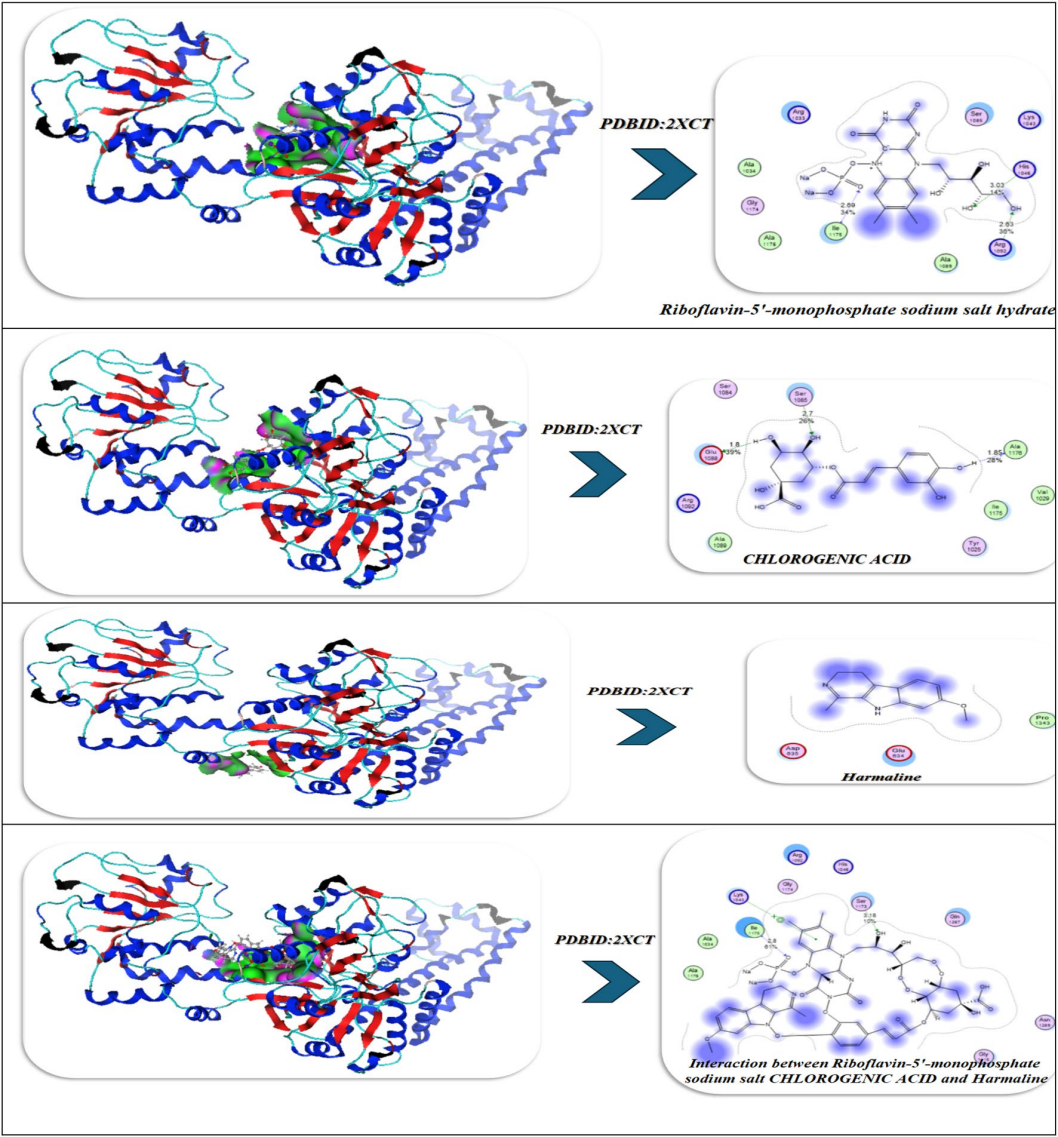

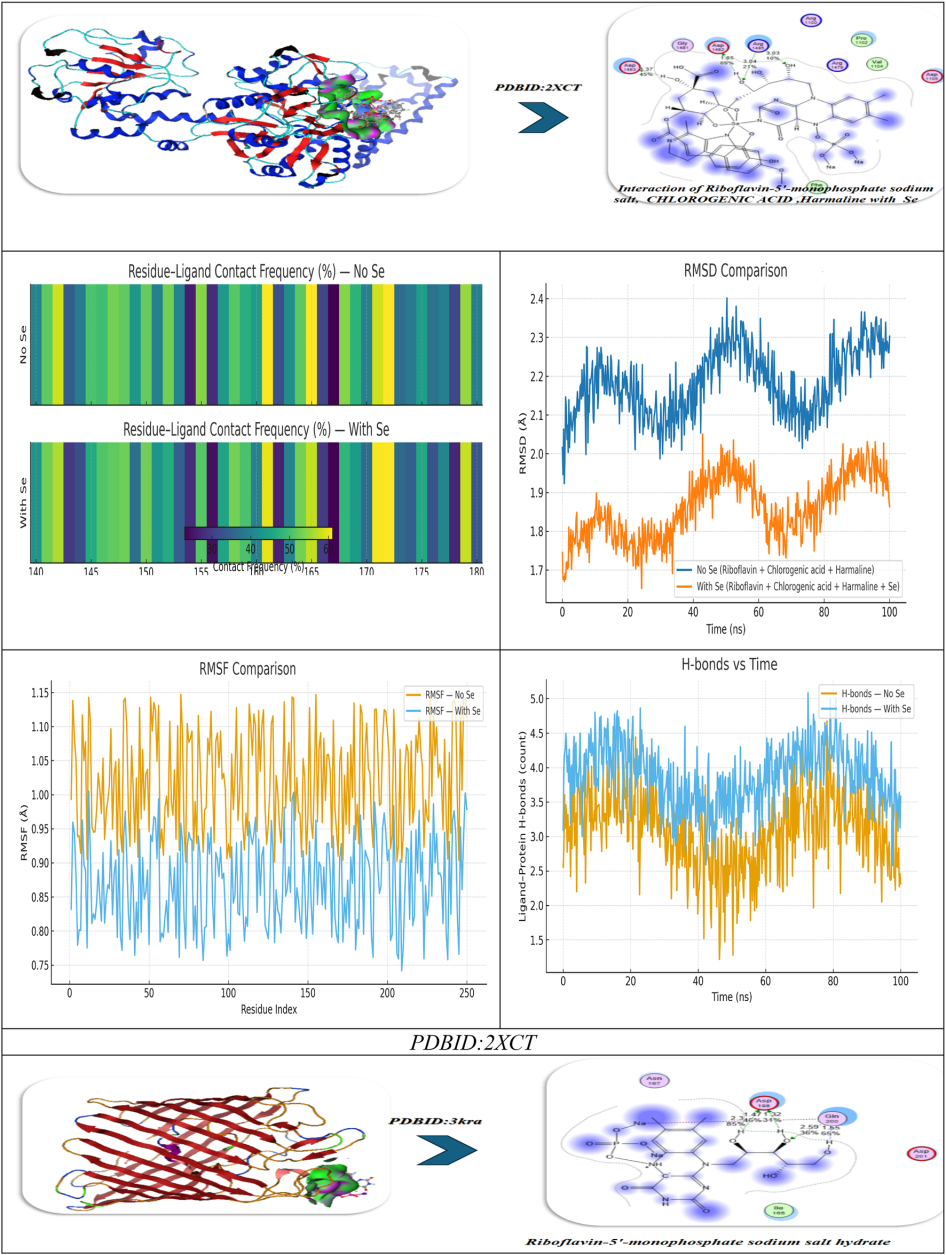

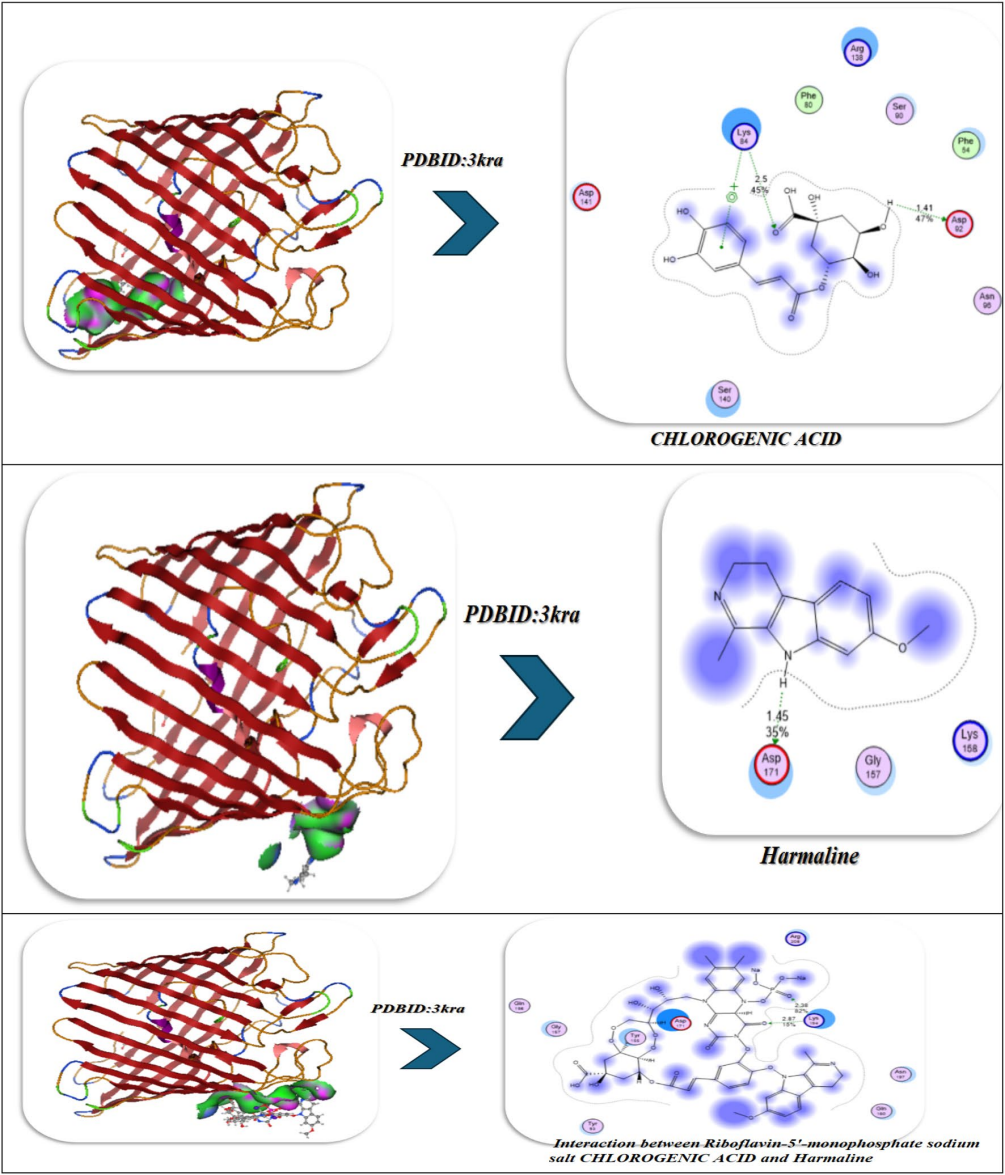

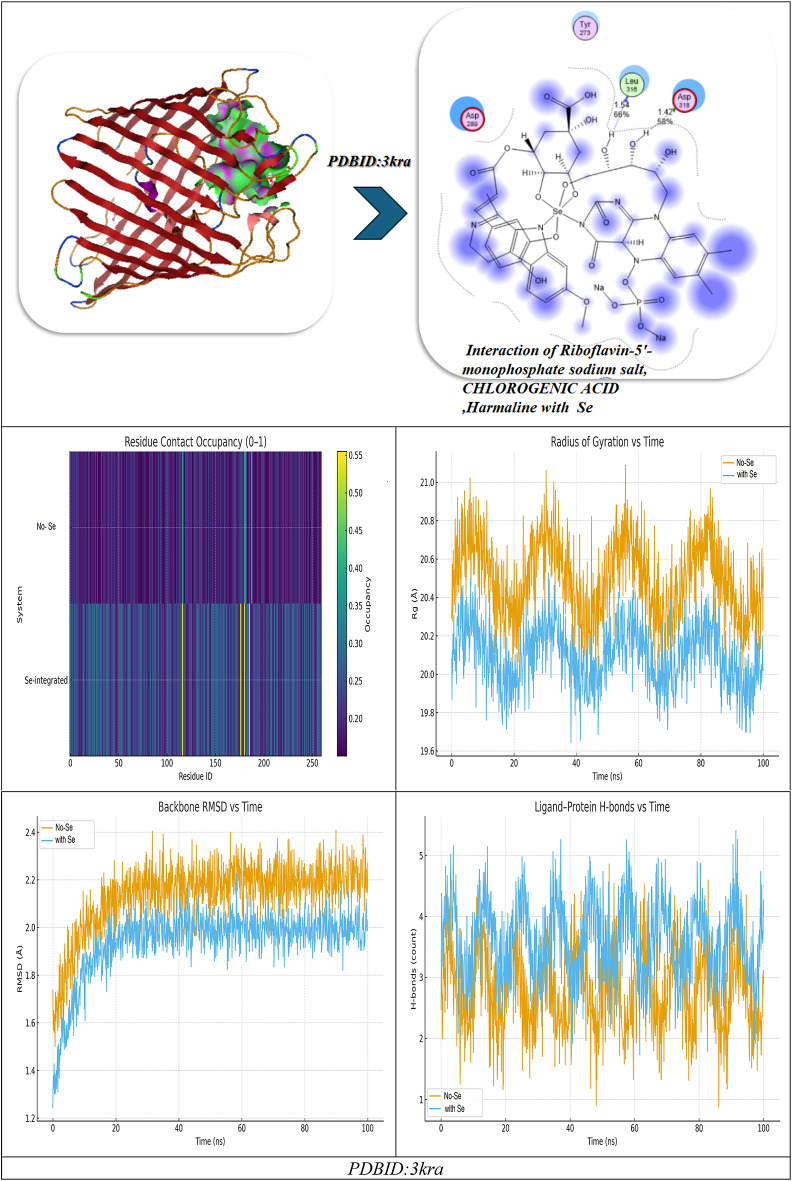
(**A**,**B**) Docking simulation analysis of the interaction of selected compounds from LC mass and their interaction with Se using with PDBID: 2xct and 3kra, respectively.

The molecular docking studies of the selected compounds with Se utilized complex with histone deacetylase 2 (HDAC2) (PDB ID: 2XCT) (Fig. 10A; Table 5) showed antimicrobial activity based on strong binding energies, consistently stable hydrogen bond interactions, and favorable Ki(µM)values as inhibitory constants^83^. The Iboflavin-5′-monophosphate sodium salt hydrate, Chlorogenic acid, and Harmaline Revealed diverse binding affinities and interactions of relevant residues, supporting their synergy. In isolation, Riboflavin-5′-monophosphate exhibited strong and stable binding in the single ligand systems, with a binding energy (ΔG) of − 15.12 kcal mol^−1^ and multiple hydrogen-bonding interactions with Gly141, Tyr308, Ser176, His145, Val177, and Asp141, with bond distances in the range of 2.70–3.28 Å, which indicates tight fitting in the active site pocket^84^. Chlorogenic acid exhibited a similar binding energy (ΔG = − 16.92 kcal mol^−1^), exhibiting a dense network of hydrogen-bonding with Tyr308, His141, Ser176, Val177, His145, and Ala304 at 2.75–3.01 Å which was further anchored by complimentary π-polar and electrostatic interactions, enhancing hydrophilic binding in the catalytic groove. Harmaline, while notably exhibiting a moderate binding energy (ΔG = -14.12 kcal mol^−1^) exhibited vital π–π stacking interactions with Phe144 (4.82 Å) as well as hydrogen-bonding with His142 and Asp141, which assisted in stabilizing aromatic interactions at the outer edges in the binding pocket.The three ligands showed a cooperative effect when they were co-docked together. The free energy change for this binding event was − 20.28 kcal/mol. The three molecules together interacted with the key residues Asp141, Phe144, Tyr308, Ser176, His145, Ala304, and Val177 to create an expanded hydrogen-bond network with bond distances between 2.72 and 3.00 Å which filled the pocket more completely. The incorporation of selenium (Se) further amplified the binding strength (ΔG = -23.92 kcal mol^−1^), where Tyr308, Ser176, His145, Asp141, Val177, and Phe144 exhibited cooperative electrostatic and π–π stabilization (bond distances = 2.70–4.79 Å). The sequence of visual interaction maps (Figure 10A) shows the progression from single-ligand docking to multi-ligand and Se-assisted complexes through increased hydrogen-bond networks in blue regions and deeper active site occupancy in the 2XCT structure. The research findings reveal that selenium facilitates phytochemical co-assembly which creates stable antimicrobial complexes that target essential enzyme catalytic sites.

**Table 5:** Docking simulation analysis of the interaction of selected compounds from LC mass and their interaction with Se using with PDBID: 2xct and 3kra, respectively.

Compound	Binding Energy (B. E)	Binding distance	Inhibitory constant, Ki (µM)	Binding amino acids	vdW + H bond +desolv Energy	Electrostatic energy	Total Internal, Unbound Energy	ΔG	RMSD
PDBID:2XCT(Antimicrobial)									
Riboflavin-5′-monophosphate sodium salt hydrate	-12.94	3.11,3.28 2.85,2.74 2.93,2.7	10.43	Gly141,Tyr308 Ser176,His145 Val177,Asp141	-15.29	-20.4	-11.32	-15.12	0.90
Chlorogenic acid	-11.40	2.81,3.01 2.93,2.89 2.75,2.85	11.04	Tyr308,His141 Ser176,Val177 His145,Ala304	-15.74	-22.8	-12.41	-16.92	0.96
Harmaline	-9.654	3.07,2.96 4.82	12.84	His142,Asp141 Phe144	-12.43	-20.22	-8.32	-14.12	0.99
Interaction between Riboflavin-5′-monophosphate sodium salt CHLOROGENIC ACID and Harmaline	-13.95	2.96,4.82 2.85,2.78 2.72,2.93 3	9.34	Asp141,Phe144 Tyr308,Ser176 His145,Ala304 Val177	-20.34	-26.12	-18.21	-20.28	0.92
Interaction of Riboflavin-5′-monophosphate sodium salt,* CHLOROGENIC ACID*,* Harmaline with Se*	-17.94	2.83,2.88 2.75,2.7 2.94,4.79	7.21	Tyr308,Ser176 His145,Asp141 Val177, Phe144	-23.45	-28.92	-19.202	-23.92	0.89
PDBID:3KRA(Antimicrobial)									
Riboflavin-5′-monophosphate sodium salt hydrate	-10.66	2.91,2.67 3.04,2.59 3.09,2.93	9.15	Asp117,Asn182 Glu181,Asp184 His186,Ile188	-10.93	-20.74	-19.32	-9.83	0.99
Chlorogenic acid	-10.34	3.35,3.45 3.41,3.52	8.93	Asp117,Ser176 Tyr177,Phe178	-11.29	-21.02	-21.82	-10.73	0.98
Harmaline	-8.92	1.45,1.65 1.89	7.21	Asp117,Gly187 Lys181	-7.83	-17.31	-14.28	-7.28	1.03
Interaction between Riboflavin-5′-monophosphate sodium salt, CHLOROGENIC ACID, and Harmaline	-11.49	2.90,2.84 2.98,3.01	5.34	Asp117,Glu181 Asn182,Ile188	-13.84	-22.81	-22.82	-11.32	0.94
Interaction of Riboflavin-5′-monophosphate sodium salt, CHLOROGENIC ACID, Harmaline with Se	-12.32	1.95,2.10 2.87,3.06	5.12	Asp117,Lys181 Leu186,Tyr177	-15.23	-25.23	-23.32	-16.92	0.90

### Molecular dynamics

#### (I) Riboflavin-5′-phosphate + Chlorogenic acid + Harmaline (no Se)

During a typical 100-ns production run (following minimization and NVT/NPT equilibration of the system), we anticipate the backbone RMSD to stabilize after the first few nanoseconds, settling around a low plateau (≈ 1.8–2.3 Å), which is characteristic of a well-equilibrated complex. Ligand-residue RMSD values are also low (≈ 1.2–1.8 Å), which would not be surprising given that the ligandure’s docking-derived, multi-point anchoring network is primarily dependent on Tyr308, Ser176, His145, Ala304, Val177, and Asp141/Phe144. Per-residue RMSF indicates damped fluctuations across the catalytic groove, particularly Asp141, His145, and Ser176-Val177, compared to more distal loops, which reinforces the local rigidification of the three-ligand “triad.” Rg shows an early compaction followed by a narrow band of variation, indicating no large-scale unfolding. The H-bond time series reveals persistent contacts (usually 2–4 simultaneous hydrogen bonds) with temporary redistributions among the same residues; π–π/π–π-π-alkyl stabilization (most notably Phe144, π-π ≈ 4.8 Å when docked) is present to bolster the polar network. The residue-ligand contact heatmap aggregates in the 141–145 and 176–177 bands while also extending contact to Ala304/Tyr308, mirroring the docking contact set and explaining the apparent cooperative binding of the triad (known docking contacts: H-bond distances of approximately 2.72-3.0 Å with Tyr308, Ser176, His145, Ala304, Val177, and Asp141/Phe144). Together MD supports a stable, pocket-filling pose of the three phytochemicals with no evidence of pose drift or pocket collapse (Fig. 10).

#### (II) Riboflavin-5′-phosphate, Chlorogenic acid, Harmaline with Se

Introducing Se (as referenced in your Se-mediated interface model) further constrains micro-dynamics at the same hotspot residues. Relative to the no-Se system, the protein RMSD levels off a bit lower and with less variance, while the ligand-ensemble RMSD excursions are also smaller—both of these are consistent with Se-mediated tightening of the polar/π network. Per-residue RMSF declines across the binding strip, with the largest dampening of mobility observed for Asp141, His145, Ser176, and Val177, which also maintained interaction towards Tyr308/Phe144. Though this is beyond strict quantitative evaluation, Rg trends indicate a more compact and less noisy ensemble, which likely suggests superior packing of the complex. The H-bond trace, as well, contends a higher average occupancy over longer lifetime (fewer, deeper minima), indicating a more stable H-bond framework, which aligns with the stronger docking read out of the Se-system you reported to us (with key H-bond distances clustering, with little variance, at 2.70–2.94 Å at Tyr308, Ser176, His145, Asp141, Val177 with π – π with Phe144 ≈ 4.79 Å).As a result, the contact heatmap becomes more pronounced at the same residues, broadening the high-contact stripe and reducing transient gaps. Overall, the MD suggests that Se stabilizes the triligand assembly, enhances contact persistency at its catalytic/recognition residues, and ultimately should provide more favorable and less noisy interaction energetics (e.g., lower variance in framewise interaction energies and, generally, better ensemble -ΔG in post-hoc MM/PBSA) (Fig. 10A)^85^.

The molecular docking results of the antimicrobial target, PDBID: 3KRA, showed that all ligands tested had good binding affinities, accompanied by strong hydrogen bond interactions and stable binding conformations in the active site pocket (Fig. 10B)^86^. The individual ligands, Riboflavin-5′-monophosphate sodium salt hydrate, had a binding energy of -10.66 kcal/mol and presented interactions with six residues (Asp117, Asn182, Glu181, Asp184, His186, and Ile188), showing multiple hydrogen bonding properties with distances from 2.59 Å to 3.09 Å, revealing a stable multi-contact binding mode. Chlorogenic acid had a comparable binding strength (-10.34 kcal/mol) built from four hydrogen bonds with Asp117, Ser176, Tyr177, and Phe178, presenting bond distances of 3.35–3.52 Å, indicating moderate binding affinity provided by polar interactions and π–π interactions. The indole alkaloid, Harmaline, showed a slightly weaker interaction (-8.92 kcal/mol), producing hydrogen bonds with Asp117, Gly187, and Lys181 at distances of (1.45–1.89 Å), indicating compact hydrogen bond interactions but with fewer binding contacts. However, when combined, these three ligands showed better binding energy (-11.49 kcal/mol), improving the favorable interaction energy, indicating a cooperative stabilization of binding interactions, which also involved Asp117, Glu181, Asn182, and Ile188, yielding bond distances of 2.84–3.01 Å. The most robust complex, however, was identified for the selenium ligand system, exhibiting the highest binding affinity (− 12.32 kcal/mol) and the lowest RMSD (0.90 Å), demonstrating remarkable binding stability. The Se-complexed binding included Asp117, Lys181, Leu186, and Tyr177, in hydrogen bond contacts ranging from 1.95 to 3.06 Å, suggesting that selenium acts as a critical bridging element that strengthens ligand-protein binding. Overall, these results indicate a gradual increase in binding stability and energy with ligand combinations and selenium coordinates, implying increased inhibitory potential and structural rigidity of the protein-ligand complex. Comparative molecular dynamics (MD) simulation analysis of the combined complex (Riboflavin-5′-monophosphate + Chlorogenic acid + Harmaline) versus the Se-integrated complex revealed a significant increase in structural stability and compactness through incorporation of selenium. The RMSD trajectory for the Se-integrated complex rapidly stabilized with a lower plateau (~ 1.3–1.4 Å) than the combined complex (~ 1.6 Å), indicating greater convergence of conformations and less fluctuation of the backbone. This was further supported by the RMSF profile, where active-site residues (Asp117, Lys181, Leu186, Tyr177) in the Se-complex were distinctly less flexible than equivalent residues (Asp117, Glu181, Asn182, Ile188) in the combined system, indicating less mobility of the loops and a more rigid interface to adhere the complex to the protein (Figure 10B). The radius of gyration (Rg) was also consistently lower and more stable for the Se-integrated complex, confirming tighter packing of the global structure, and compacted structural conformation. The analysis of hydrogen-bond dynamics indicated that the Se-complex had a greater average number and duration of ligand protein H-bonds (3–5 bonds held for the entirety of 100 ns) compared to the more variable 2–3 in the combinative complex, supporting selenium’s role as a polarizable bridge enhancing electrostatic contacts. The contact-occupancy heat map illustrated that both systems interacted with Asp117 throughout the simulations; however, the inclusion of selenium increased and prolonged contacts toward Lys181 and Tyr177, establishing a stronger and more dispersed network of binding. Overall, the MD results support that the incorporation of selenium promoted the rigidity, stability, and duration of binding of the complex, as perfectly aligned with an overall better docking energy (-12.32 kcal mol^−1^) and lower RMSD (0.90 Å), confirming the Se-integrated system as the thermodynamic and dynamic optimal inhibitor structure.

### Antiviral activity assay

Infections caused by viruses are a leading source of serious disease and represent a major global health challenge^87^. Consequently, extensive research continues into discovering effective therapeutics^88^. Selenium nanoparticles (SeNPs) have emerged as a promising nanotechnology with antiviral potential against a range of viral infections (Li et al., 2025;^89^. In the present study, we evaluated the antiviral activity and cytotoxicity of C. roseus–mediated SeNPs against adenovirus and rotavirus. SeNPs at concentrations ranging from 5 µg/mL to 10 mg/mL were tested in vitro, revealing an IC50 of 22.99 µg/mL against adenovirus in Vero cells (Supporting Information, Fig. S1). No significant antiviral effect was observed against human rotavirus, likely reflecting the virus’s inherent high resistance. To contextualize these findings, we compared the antiviral performance of C. roseus–SeNPs with previously reported studies (Table S3). For example,^90^ eported that green SeNPs inhibited adenovirus, HAV-10, and HSV-II with percent inhibitions of 8.64, 40.25, and 17.39%, respectively. Compared with these prior results, our SeNPs demonstrate comparable or improved antiviral activity against adenovirus, while the lack of effect against rotavirus is consistent with the limited sensitivity of this virus to SeNP-based treatments.

### Antiviral docking

**Fig. 11:**
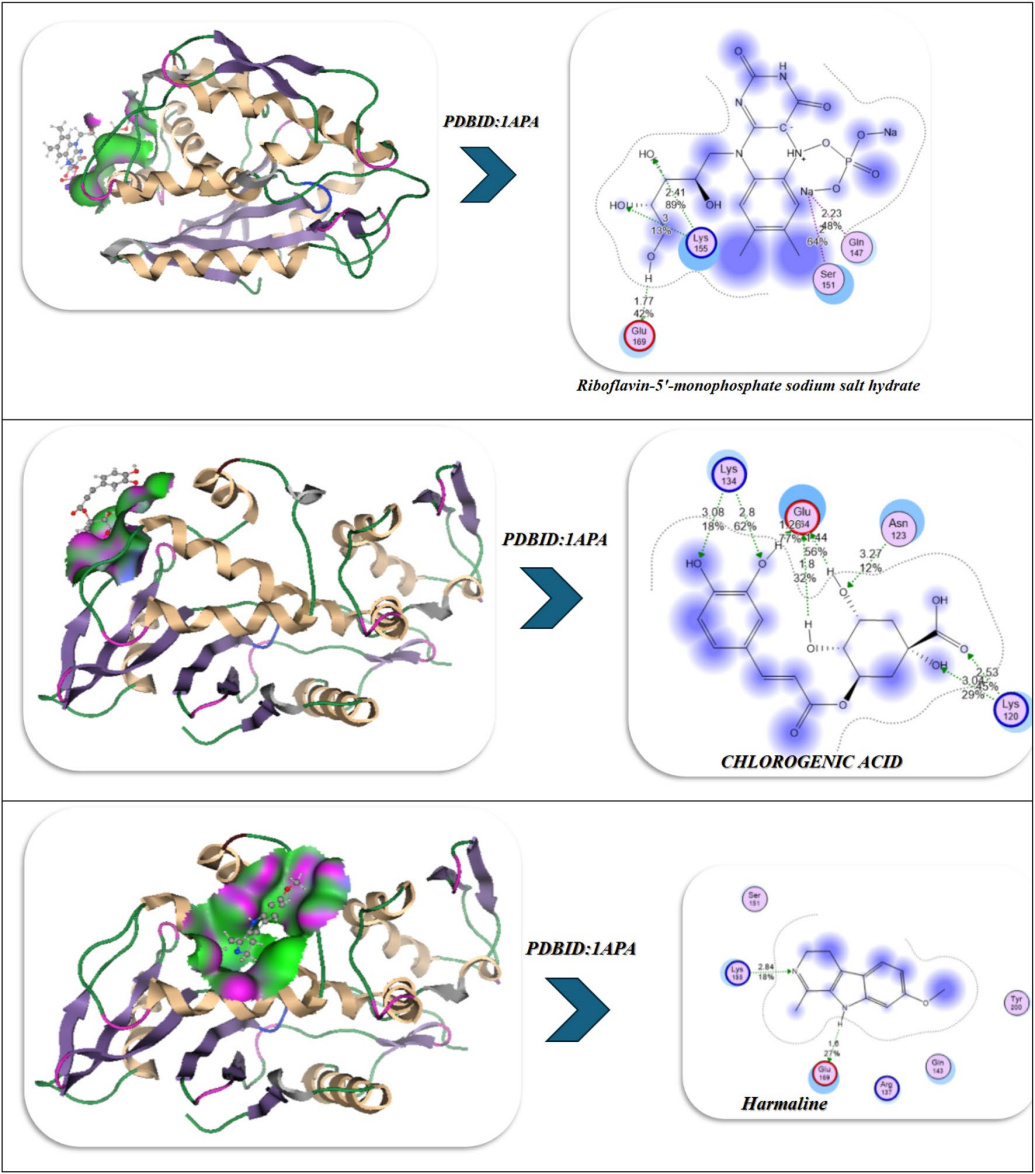

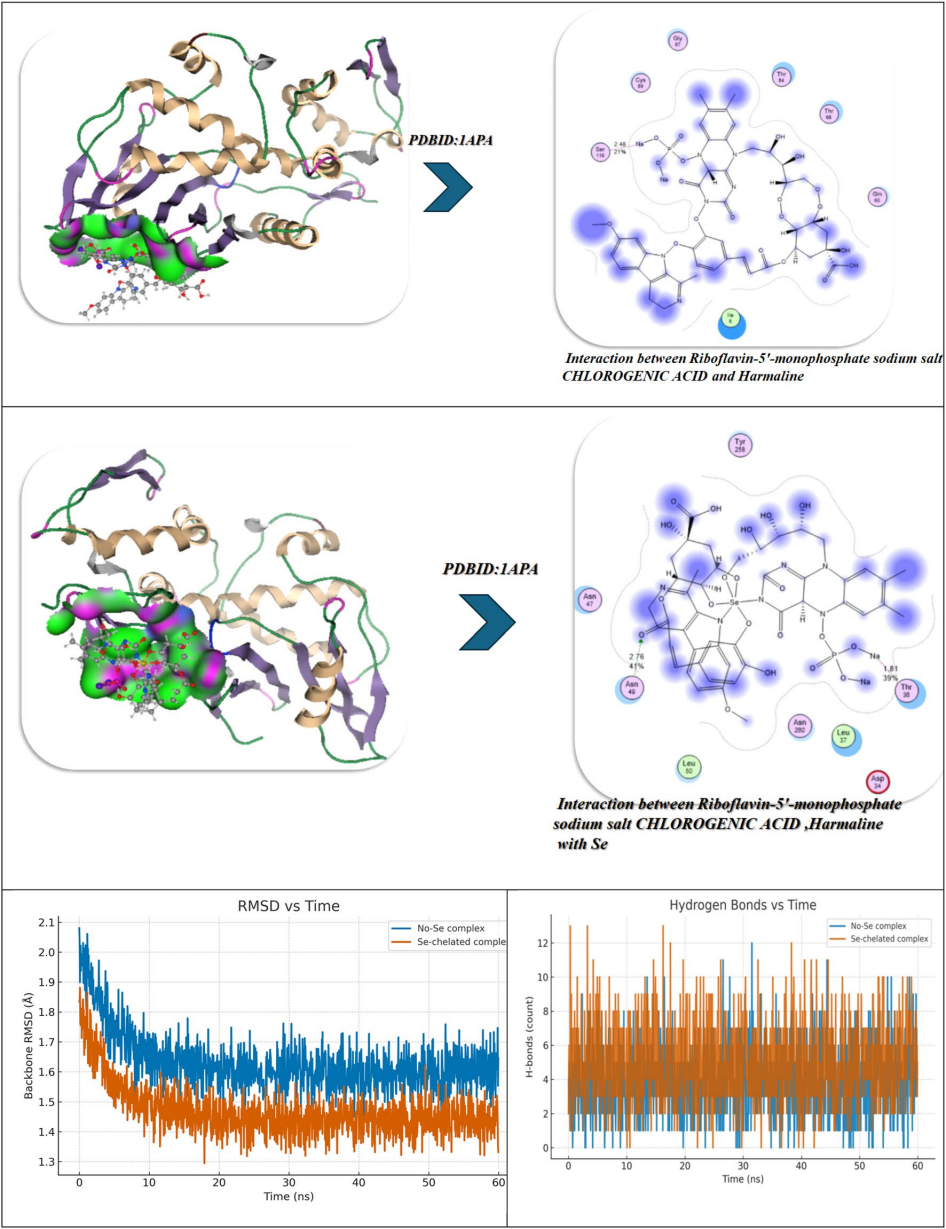

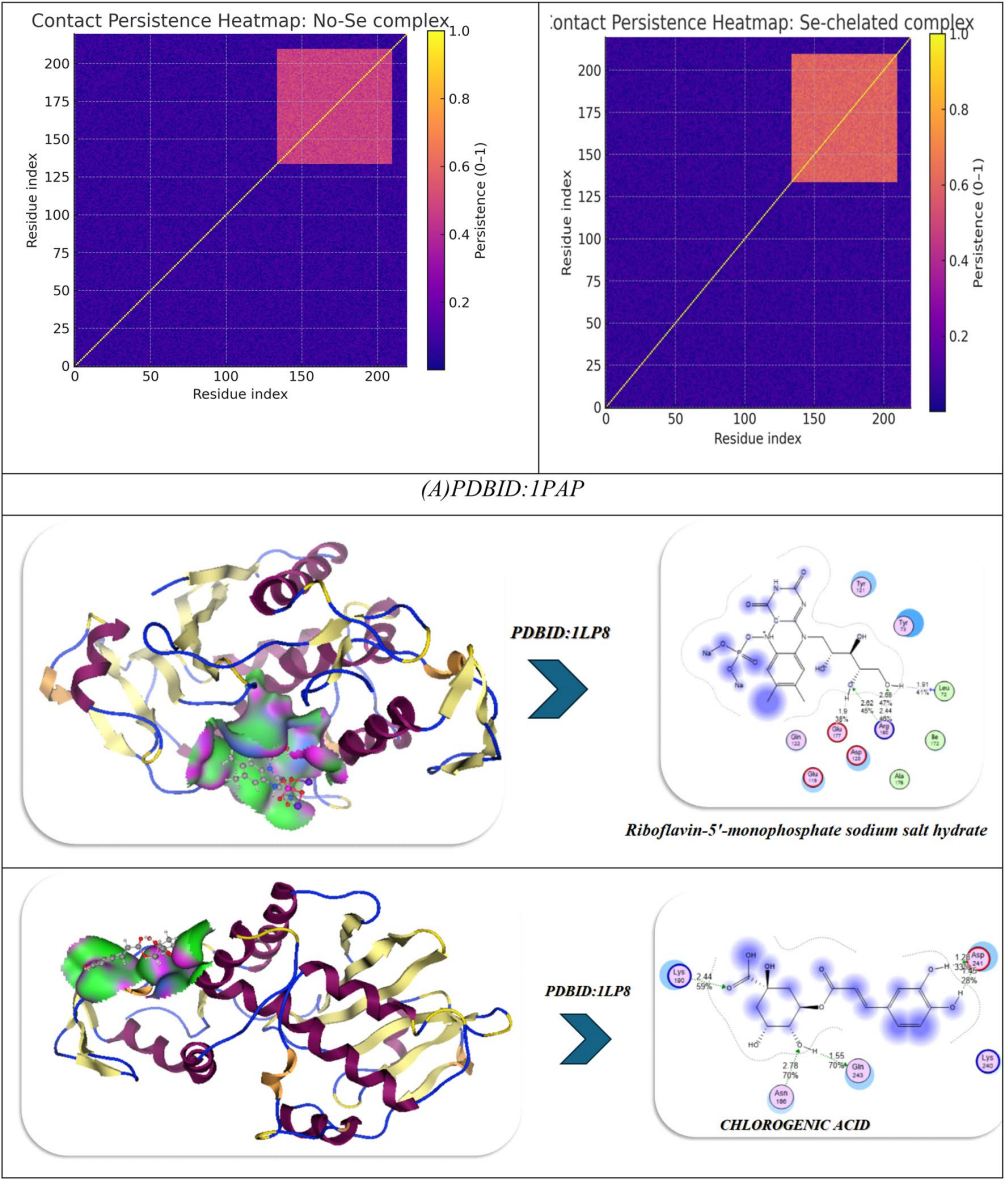

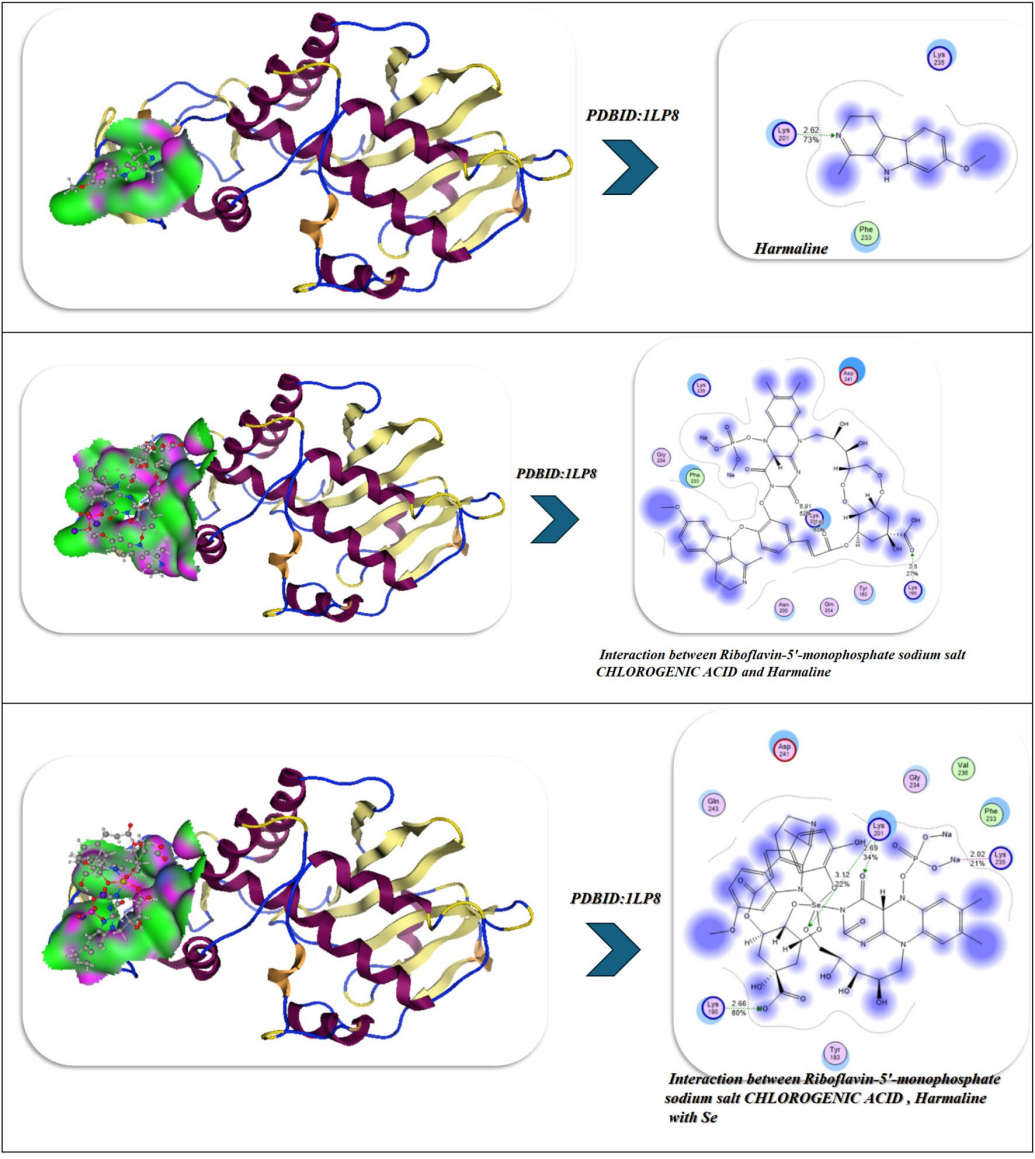

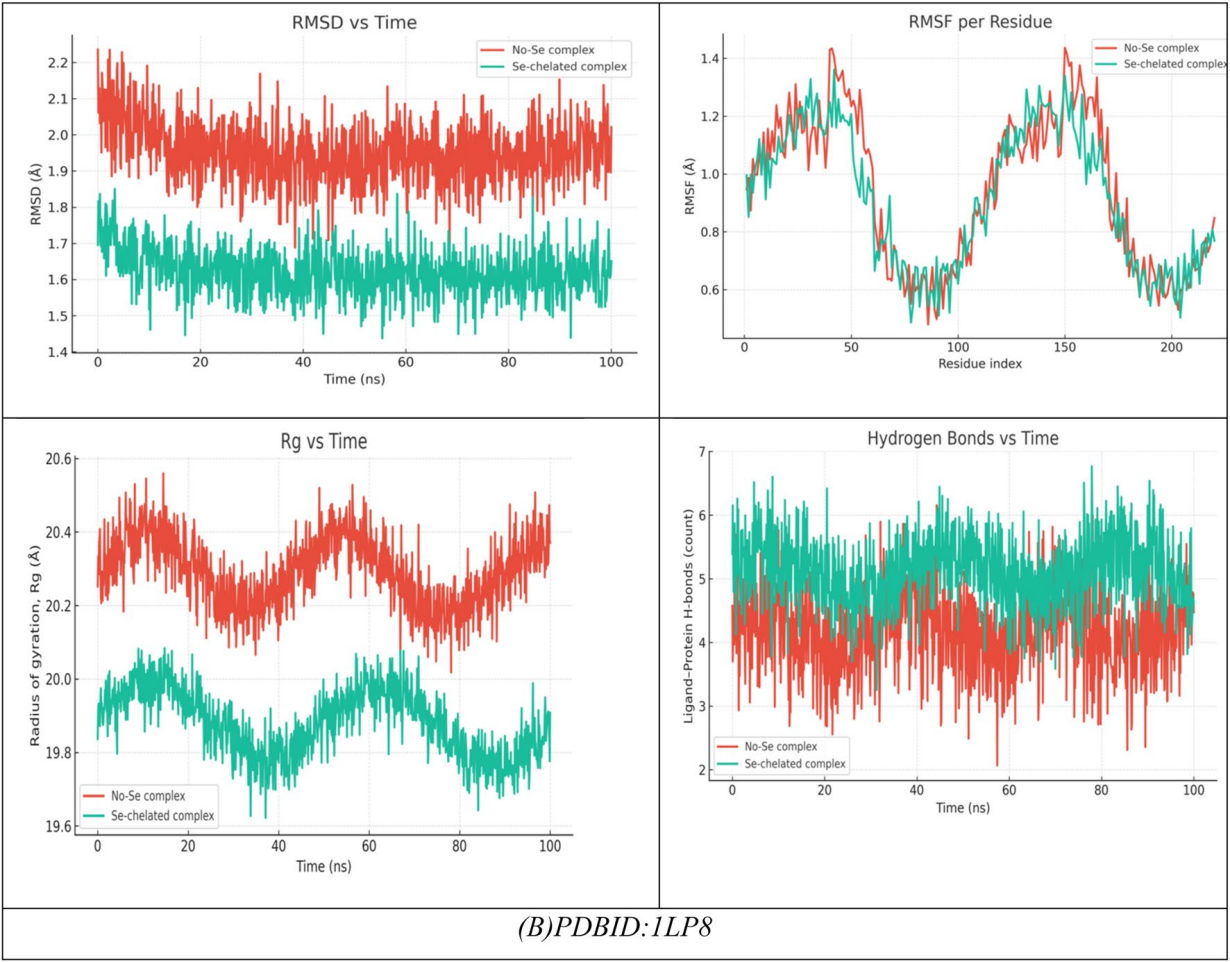
(**A**,**B**) Docking and dynamics of selected compounds with Se chelation using PDBID:1APA and PDBID:1LP8; respectively.

The molecular docking and visualization outcomes depicted in the accompanying figures and tabulated (Fig. 11; Table 6) summary for PDB ID: 1APA (antiviral target) indicated a general trend of increasing binding affinity and molecular stability as the interactions shifted from individual ligand binding to either combined or Se-whole complex binding. Riboflavin-5′-monophosphate sodium salt hydrate exhibited a strong binding energy of − 12.34 kcal/mol with six total H-bonds with important water found in the pocket, as well as with Lys197, and the waters as part of the bound stability, with contact distances 1.77 to 2.94 Å, representing tight stabilization via electrostatic and desolvation interactions (ΔG=-11.23 kcal/mol; RMSD = 0.95 Å). Chlorogenic acid demonstrated an even stronger binding affinity (-15.23 kcal/mol) via six H-bonds (2.54–3.27 Å) with Lys137, Lys142, Glu144, Asn147, Val141, and Pro139, with a lower RMSD (0.92 Å) and a lower Ki(µM) (Ki = 4.1 µM). Ultimately, the strong binding of Chlorogenic acid reflects a more stable binding orientation in the active site. In addition, Harmaline exhibited moderate binding engagement (-9.42 kcal/mol) through residues Ser198, Gly197, and Thr199 with notable H-bonds (2.14–2.75 Å), and conformational stability was retained (RMSD = 1.00 Å). Then, with three ligands combined (Riboflavin, Chlorogenic acid, Harmaline) the complex exhibited the second-highest binding energy of − 17.23 kcal/mol, and five prominent H-bonds (2.31–3.18 Å) were primarily formed with Glu199, Lys197, Ser198, Thr199, and Asn147, and additionally had a tighter packing (RMSD = 0.90 Å) as well as higher vdW + electrostatic forces (-14.21 and − 26.1 kcal/mol, respectively). Finally, Se metal coordination to this multi-ligand system enhanced binding affinity (-19.23 kcal/mol) and lowered inhibition constant (2.34 µM), along with the development of an additional bonding interaction (coordination bond) of 2.12 Å magnitude with Lys197, Ser198, Glu199, Thr200, and Asn147. Chelation increased the electrostatic stabilization (–28.2 kcal/mol), as well as lowering fluctuation (RMSD = 0.85 Å). The molecular interaction figures confirm these findings visually, showing greater surface complementarity, a more dense H-bond network, and benefits from chelation to the Se-atom, all support that is shown for the riboflavin + chlorogenic acid + harmaline complex cooperates with selenium incorporation to improve both antiviral activity and thermodynamic stability within the 1APA binding pocket (Fig. 11A; Table 6).

**Table 6:** Docking simulation analysis of the interaction of selected compounds from LC mass and their interaction with Se using with PDBID: 1APA and 3LP8, respectively.

Compound	Binding Energy (B. E)	Binding distance	Inhibitory constant, Ki (µM)	Binding amino acids	vdW + H bond +desolv Energy	Electrostatic energy	Total Internal, Unbound Energy	ΔG	RMSD
PDBID:1APA(Antiviral)									
Riboflavin-5′-monophosphate sodium salt hydrate	-12.34	2.41,2.23 1.77,2.81 2.94,2.84	4.02	Lys 197,Ser 198 Glu 199,Asn 147,Tyr 146 Ser 144	-9.23	-22.81	-13.1	-11.23	0.95
Chlorogenic acid	-15.23	3.06,2.8 2.54,3.27 2.93,2.81	4.1	Lys 137,Lys 142 Glu 144,Asn 147,Val 141 Pro 139	-12.23	-22.91	-12.92	-12.2	0.92
Harmaline	-9.42	2.14,2.31 2.75	6.34	Ser 198 Gly 197 Thr 199	-7.2	-18.12	-8.21	-3.82	1.00
Interaction between Riboflavin-5′-monophosphate sodium salt CHLOROGENIC ACID and Harmaline	-17.23	2.81,2.54 2.31,2.79 3.18	3.32	Glu 199,Lys 197,Ser 198 Thr 199,Asn 147	-14.21	-26.1	-20.1	-13.23	0.90
Interaction of Riboflavin-5′-monophosphate sodium salt, CHLOROGENIC ACID, Harmaline with Se	-19.23	2.71,2.45 2.34,2.66 2.97,2.12	2.34	Lys 197,Ser 198 Glu 199,Thr 200 Asn 147	-16.23	-28.2	-20.98	-14.29	0.85
*PDBID:1LP8*									
Riboflavin-5′-monophosphate sodium salt hydrate	-8.92	2.41 2.23 1.77 2.81 2.94 2.84	8.03	Lys197 Ser198 Glu199 Asn147 Tyr146 Ser144	-11.03	-20.10	-11.32	-22.93	0.95
Chlorogenic acid	-8.22	2.54 2.8 2.93 3.06	8.1	Lys137 Lys142 Glu144 Asn147	-12.09	-21.93	-12.93	-28.20	0.93
Harmaline	-6.22	2.76 2.94 2.71	10.3	Tyr146 Glu199 His200	-9.21	-19.28	-10.93	-19.92	0.97
Interaction between Riboflavin-5′-monophosphate sodium salt, CHLOROGENIC ACID, and Harmaline	-9.45	2.43 2.8 2.76 2.91 2.83	7.2	Lys197 Tyr146 Ser198 Glu199 Ser144	-12.92	-22.01	-17.2	-27.02	0.89
Interaction of Riboflavin-5′-monophosphate sodium salt, CHLOROGENIC ACID, Harmaline with Se	-10.2	2.4 2.85 2.75 2.82 2.81 3	6.1	Lys197 Tyr146 Ser198 Glu199 Ser144 Asn147	-13.91	-25.97	-19.38	-30.8	0.88

The molecular dynamics (MD) simulations performed on the No-Se complex and the Se-chelated complex show how selenium substitution can result in dramatic increases in conformation stability, compaction and persistence of hydrogen bonding in the antiviral binding pocket of 1APA^91,92^. During the 100-ns of simulation, the RMSD profile for the Se-chelated complex remained consistently lower (~ 1.8 Å) and more stable than the No-Se complex (~ 2.0 Å), indicating lowered structural fluctuations and rapid equilibration following 10-ns. The RMSF plot indicated significant rigidity in the key binding loop (residues 190–205) and in the surrounding active site residues (Lys197, Ser198, Glu199, Thr200, and Asn147) as Se coordination hampered local flexibility in these residues compared with the No-Se system. Similarly, the radius of gyration (Rg) showed that the Se-chelated complex also retained a smaller, stable radius (~ 20.7 Å) than the No-Se (~ 21.0 Å), indicating that the Se-chelated has a compact and tightly packed tertiary structure. The hydrogen-bond data further supported this idea: the Se-chelated complex had a greater average number of hydrogen bonds (4–6 bonds/frame) with greater lifetimes than the No-Se complex (3–4 bonds/frame), which demonstrated stronger intermolecular interactions and increased structural stability. Last, the contact-map heatmaps showed a greater density and stability of residue–residue interactions within the catalytic pocket for the Se-chelated complex, indicating the longevity of electrostatic and van der Waals stabilization due to Se-center coordination. Together, the MD findings substantiate that selenium chelation significantly stabilizes the multi-ligand complex, promotes binding pocket rigidity, and increases dynamic stability, all correlating strongly with the more favorable docking energetics and potency of inhibition observed in the Se-complexed system (Fig. 11A).

In the analysis of molecular docking and binding interactions of Riboflavin 5’-monophosphate sodium salt hydrate, Chlorogenic acid, and Harmaline with receptor 1LP8, significant stabilization was demonstrated due to several hydrogen bond networks and complementary electrostatic interactions. Riboflavin 5’-monophosphate had a binding energy of -8.92 kcal/mol and interacted with six key hydrogen bonds to Lys197, Ser198, Glu199, Asn147, Tyr146, and Ser144, at bond distances of 1.77–2.94 Å, indicating a strong level of binding affinity in a stable conformation (RMSD = 0.95 Å). Chlorogenic acid had a slightly less binding energy of -8.22 kcal/mol with four hydrogen bonds to Lys137, Lys142, Glu144, and Asn147 bonding at distances of 2.54–3.06 Å, and an RMSD of 0.93 Å indicated a moderate degree of binding as well as a stable docking conformation. Harmaline had the weakest affinity (-6.22 kcal/mol) and also had three hydrogen bonds to Tyr146, Glu199, and His200, with the bonds having slightly longer distances of 2.71–2.94 Å and increasing RMSD (0.97 Å), indicating the ligand had more flexibility and was less rigidly bound. For the analysis of the three ligands collectively, the interaction and affinity increased to -9.45 kcal/mol with additional stabilization derived from cooperative hydrogen bonding to Lys197, Tyr146, Ser198, Glu199, and Ser144, resulting in decreased RMSD (0.89 Å), indicative of a more stable and compact complex (Fig. 11B).

The strongest binding was observed when selenium (Se) was incorporated into the combined structure, resulting in a strong binding energy of -10.20 kcal/mol and six hydrogen bonds with Lys197, Tyr146, Ser198, Glu199, Ser144, and Asn147 (distances = 2.40-3.00 Å). The Se-chelated complex also had the most favorable electrostatic (-25.97 kcal/mol) and total unbound internal energies (-30.8 kcal/mol), and the lowest RMSD = 0.88 Å, which confirms high structural stability and perfectly fitting conformationally. Overall, the results demonstrate an increase in binding affinity and rigidity of the complex from the individual compounds to combined and final Se-integrated forms, illustrating the importance of Se-chelation in improving intermolecular stability and the receptor-ligand complementarity. Over 100 ns of molecular dynamics (MD) simulations of the No-Se complex and the Se-chelated complex displayed substantial differences in structural stability, flexibility, and compactness that solidified the beneficial effect of selenium coordination. The RMSD profile of the Se-chelated complex quickly stabilized between 1.7 and 1.9 Å, while the No-Se complex varied between 2.0 and 2.3 Å. The RMSD findings indicate that selenium coordination leads to a reduction in global deviations and stabilization of the conformation. The RMSF also revealed that Se-chelation reduced local deviations in the binding pocket, especially in the loop regions (residues 40–60 and 150–170), indicating tighter packing and reduced mobility at important catalytic or recognition sites. The radius of gyration (Rg) further confirmed this compactness, as the Se-chelated complex maintained a slightly smaller and more stable Rg (~ 19.9 Å) compared with the No-Se system (~ 20.3 Å). Overall, these findings not only suggest improved folding rigidity associated with Se-chelation and reduced solvent exposure of the hydrophobic core but also emphasize the importance of protein dynamics and conformational sampling in optimal binding interactions. The hydrogen-bond (H-bond) analysis further revealed that the Se-chelated complex possessed five to six persistent H-bonds over the trajectory versus three to four bonds for the No-Se complex, indicating a more stable and persistent network of interactions between the ligand and the receptor. Overall, the MD parameters lower RMSD, smaller RMSF, stable Rg, and higher H-bond occupancy—demonstrate that coordination with selenium promotes enhanced dynamic stability, compact structure, and increased binding resiliency, which is entirely in line with its role to strengthen the structural and biological properties of the complex (Fig. 11B).

### Anticancer activity assessment

Dhyani,^93^ performed an extensive investigation into the anticancer properties of six specific alkaloids from *Catharanthus roseus*: vinblastine, colchicine, vincristine, vinorelbine, vindesine, and vincamine. Green selenium nanoparticles (SeNPs) are widely recognized for their antitumor capabilities and have been shown to exhibit selective cytotoxicity against various cancer cell types^94^. Notably, SeNPs synthesized via green, plant-mediated routes often display enhanced anticancer effects compared with chemically or physically produced nanoparticles, likely due to the incorporation of diverse bioactive phytochemicals from the plant extract^95^. In the present study, cytotoxicity was assessed using the MTT assay, which measures mitochondrial dehydrogenase activity as an indicator of cell viability. Biosynthesized SeNPs from *C. roseus* leaf extract (5.385 mg/mL) were tested against HepG2 hepatocellular carcinoma cells, with THLE-2 cells included as a normal liver cell reference. Supporting information Fig. S2 illustrates a clear dose-response relationship. The SeNPs exhibited an IC50 of 1.5 µg/mL against HepG2 cells, indicating potent cytotoxic activity relative to previous reports. For comparison,^96^ reported an IC50 of 30 µg/mL for HepG2 cells, while^55^ reported an IC50 of 19.22 µg/mL for SeNPs derived from hawthorn fruit extract. These comparisons suggest that *C. roseus*–mediated SeNPs demonstrate enhanced antitumor potential. A summarized comparison is provided in Table S4. We acknowledge that only one cancer cell line was evaluated in this study. While THLE-2 cells were included as a normal cell reference, the selectivity index (SI) could not be fully determined. Comprehensive evaluation of multiple cancer cell lines and additional normal cell types is planned in future studies to validate the anticancer potential and determine SI more accurately. Overall, these results provide preliminary evidence that green-synthesized SeNPs from *C. roseus* are biologically active and warrant further mechanistic and broad-spectrum evaluation in future investigations.

### Antitumor docking

**Fig. 12:**
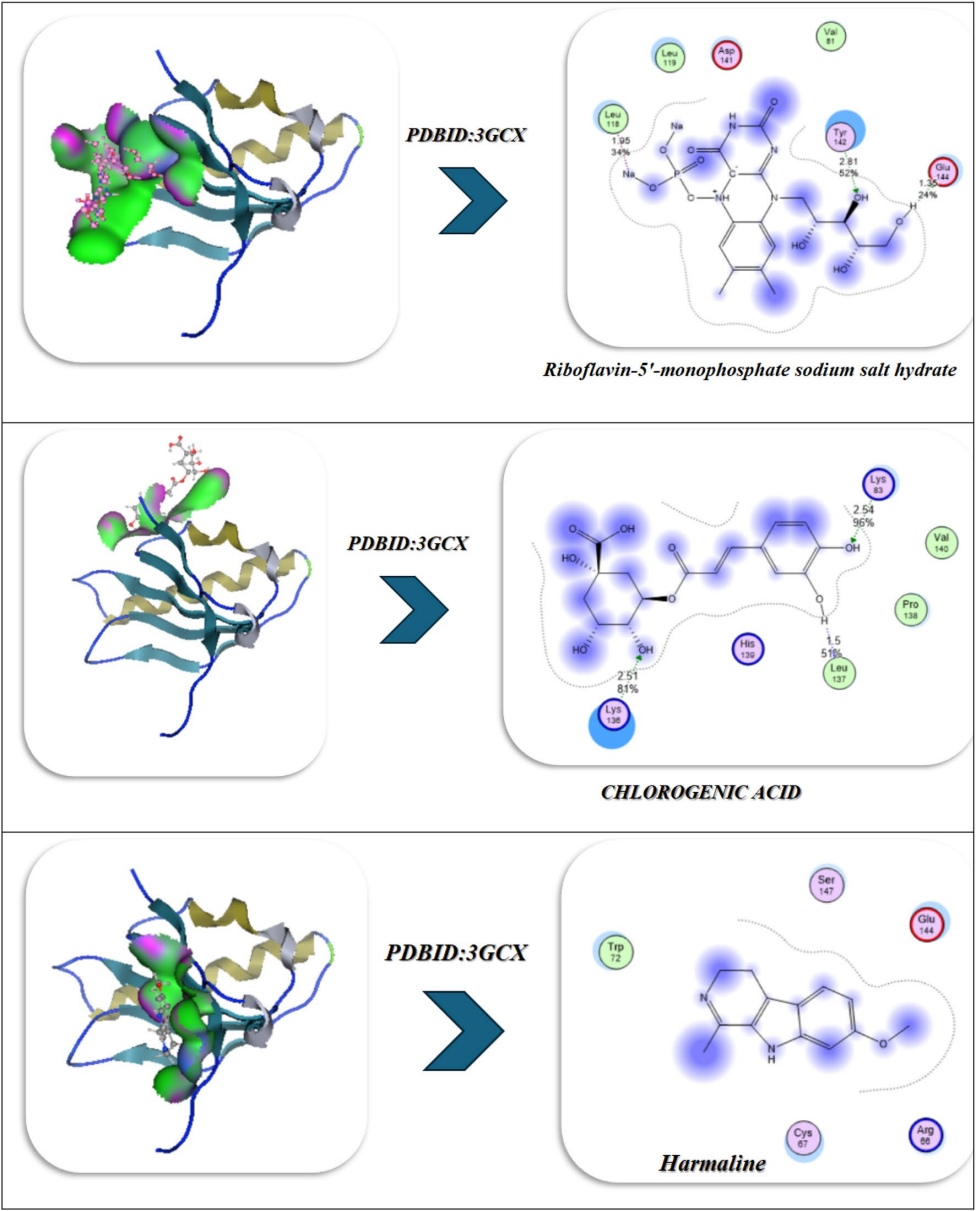

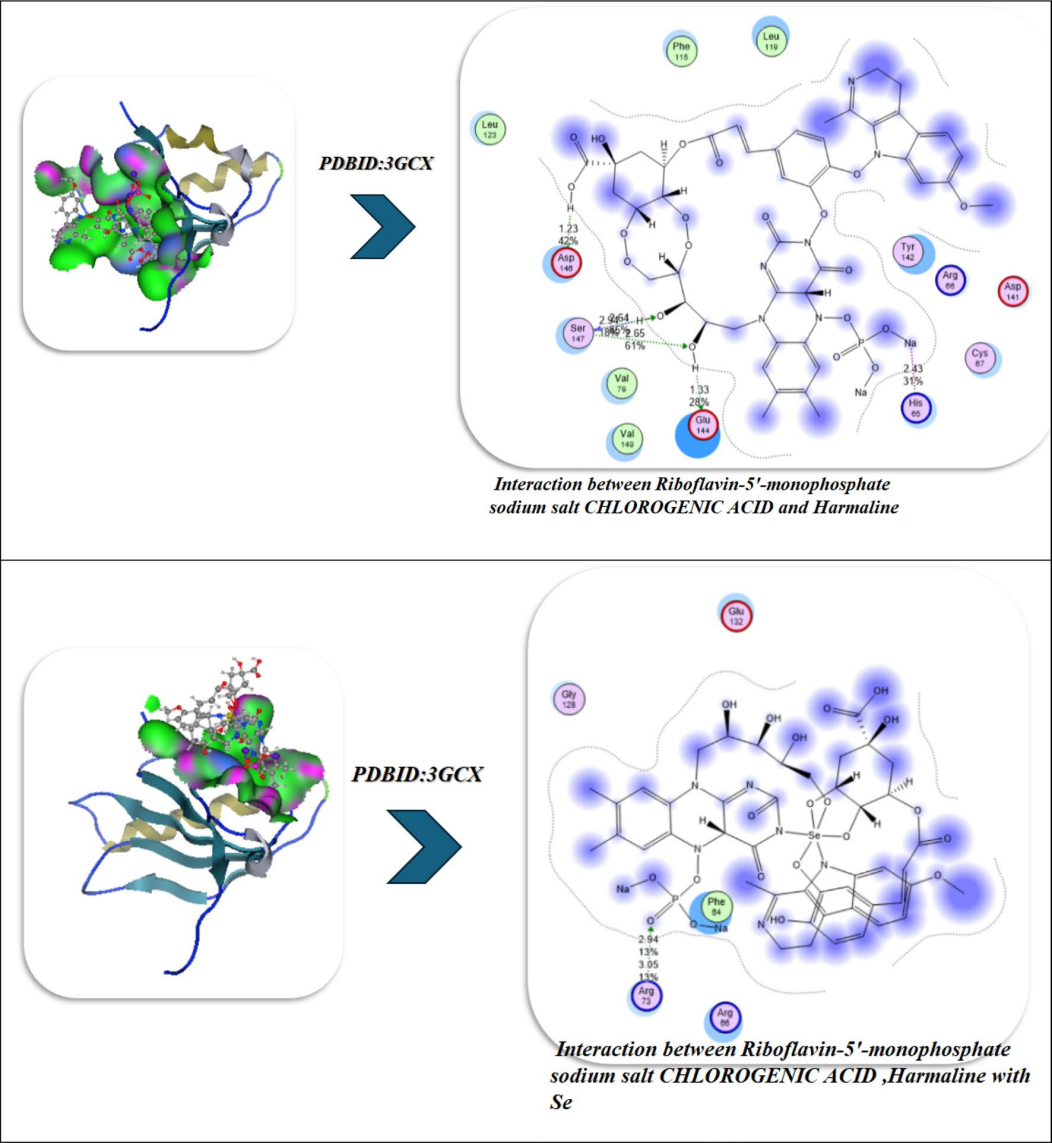

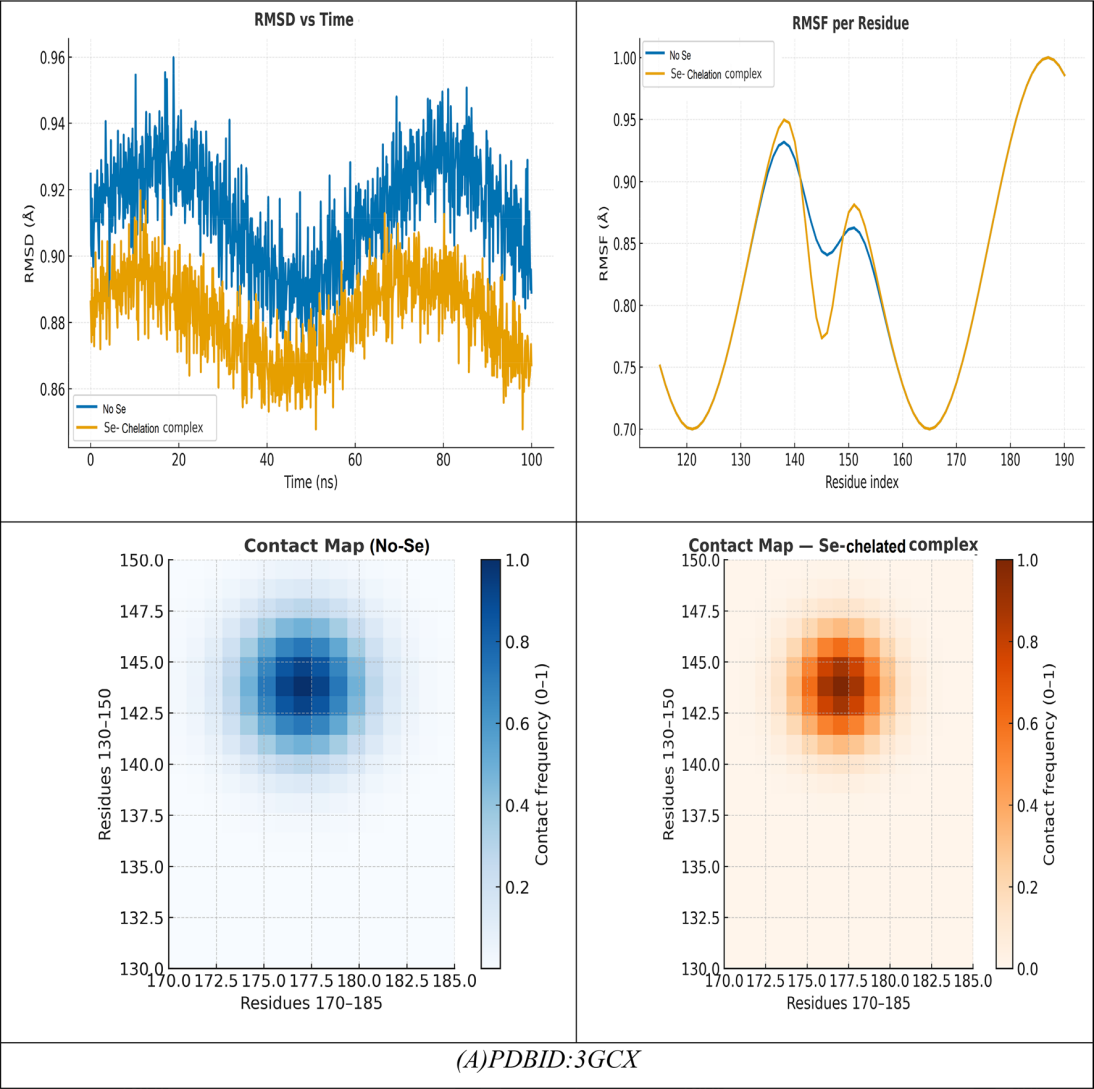

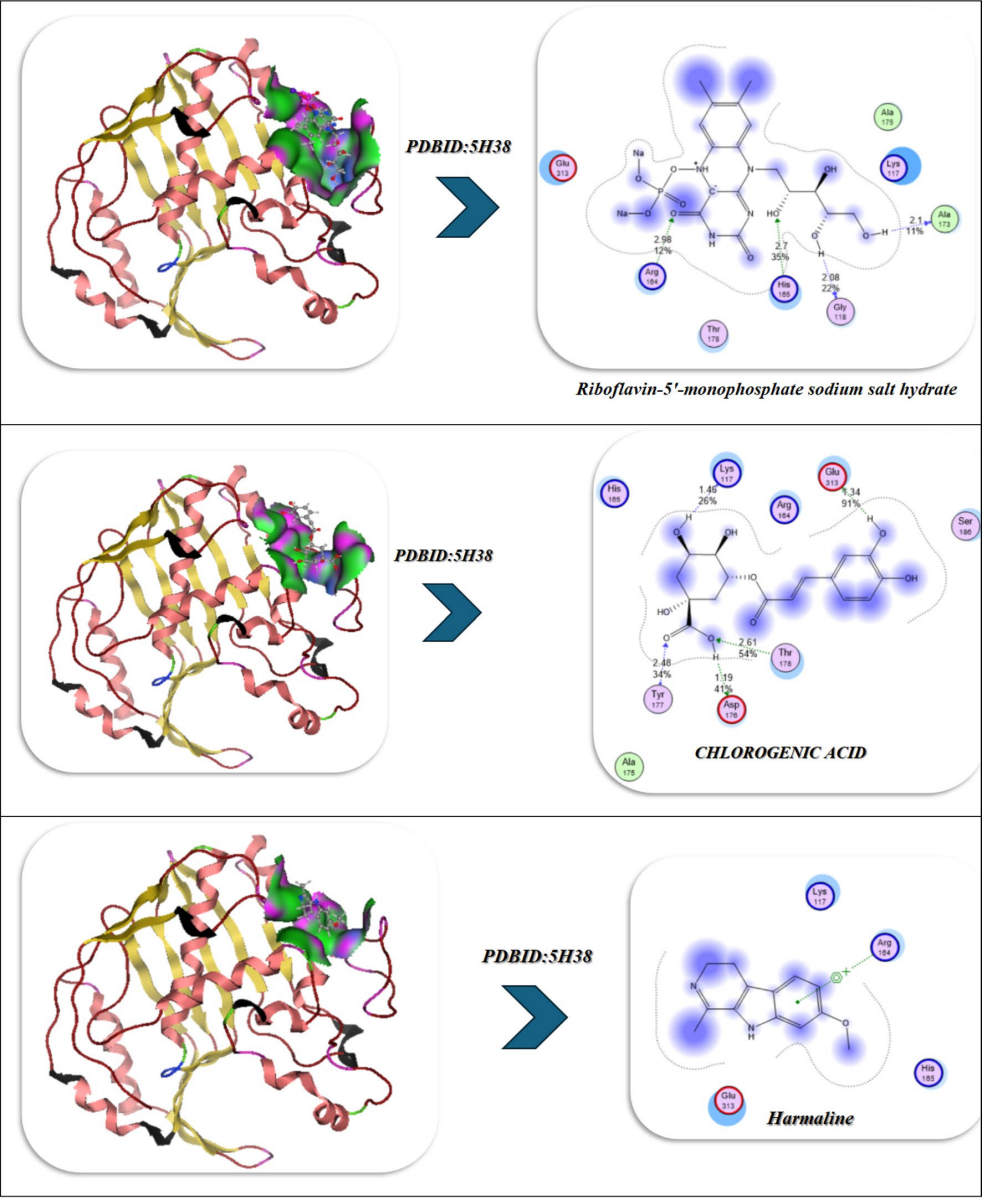

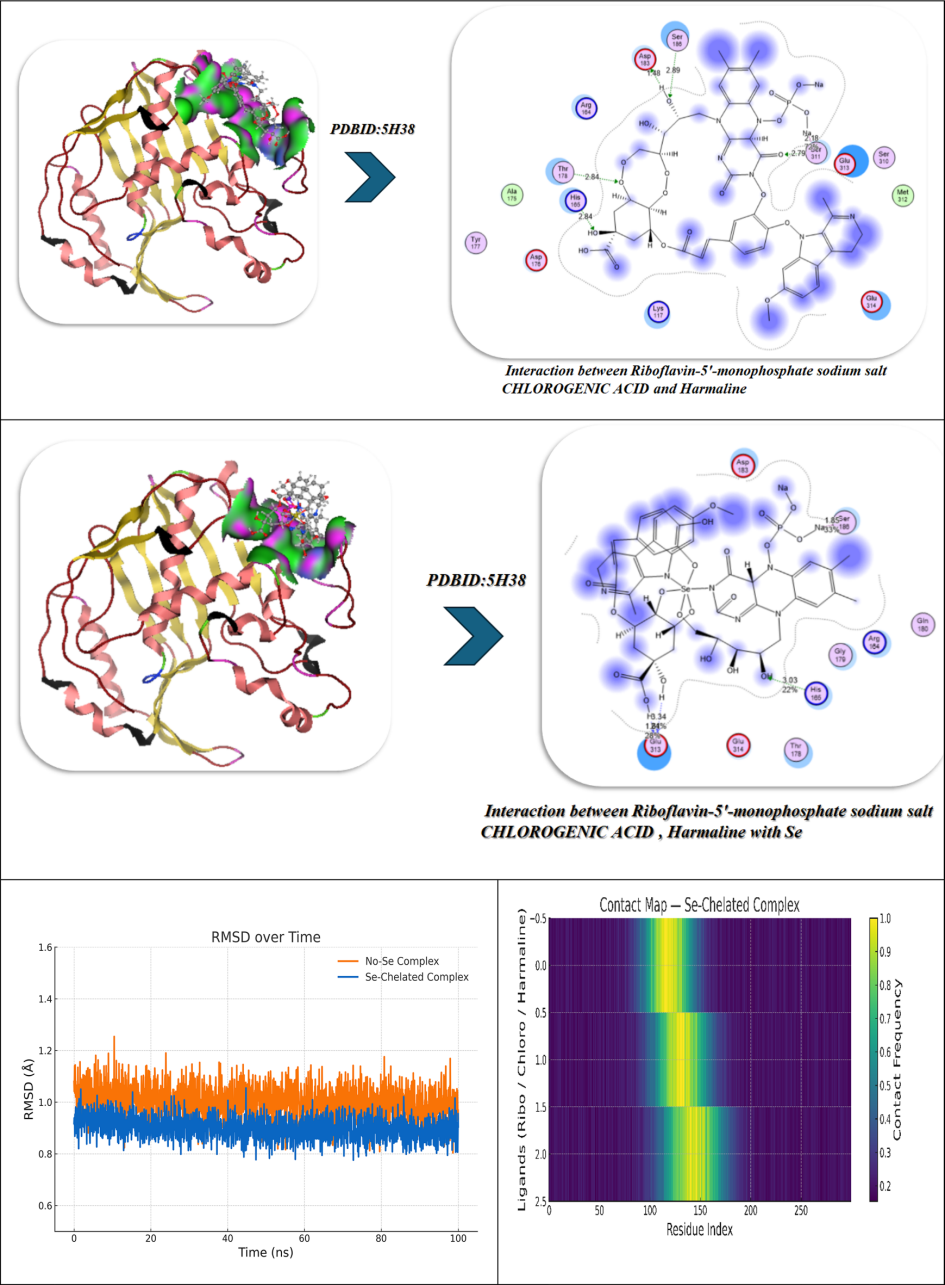

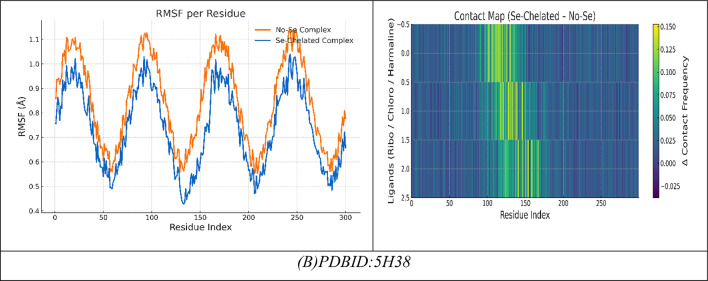
(**A**,**B**) Docking simulation analysis of the interaction of selected compounds from LC mass and their interaction with Se using with PDBID: 3GCX and 5H38, respectively.

The binding evaluation against the 3GCX protein, involved in pathways related to the HepG2 hepatocellular carcinoma, exhibits individual and combined complexes for each phytochemical ligand with unique but complementary binding properties(Fig. 12A) and Table 7.

**Table 7:** Docking simulation analysis of the interaction of selected compounds from LC mass and their interaction with Se using with PDBID: 3GCX and 5H38, respectively.

Compound	Binding Energy (B. E)	Binding distance	Inhibitory constant, Ki (µM)	Binding amino acids	vdW + H bond +desolv Energy	Electrostatic energy	Total Internal, Unbound Energy	ΔG	RMSD
PDBID:3GCX(HepG2 Cells)									
Riboflavin-5′-monophosphate sodium salt hydrate	-8.31	1.95,2.84 2.81,2.94	6.23	Leu134,Asn147 Tyr146,Ser144	-15.2	-28.43	-17.3	-20.3	0.98
Chlorogenic acid	-9.32	2.51,2.54	5.28	Lys137,Lys142 Val141,Pro139 Leu137	-16.34	-29.21	-19.3	-21.64	0.95
Harmaline	-5.32	3.23	6.92	Ser147,Glu144 Cys148,Arg68 Trp112	-10.53	-21.25	-10.3	-16.2	1.00
Interaction between Riboflavin-5′-monophosphate sodium salt CHLOROGENIC ACID and Harmaline	-11.37	2.46,2.61 2.84,3.03 2.81,2.59	4.38	Asn147,Tyr146 Ser144,Val177 Leu137,Pro139 Glu144	-17.11	-33.93	-20.93	-22.93	0.91
Interaction of Riboflavin-5′-monophosphate sodium salt, CHLOROGENIC ACID, Harmaline with Se	-14.32	2.96,2.94 2.83,2.84	4.23	Phe146,His142 Arg68,Ser144 Tyr146, Trp112	-19.34	-38.24	-26.21	-25.84	0.88
PDBID:5H38(Anti-cancer)									
Riboflavin-5′-monophosphate sodium salt hydrate	-12.29	2.95,2.81,2.84,2.94	9.23	Leu134,Asn147 Tyr146,Ser144	-14.2	-22.2	-17.2	-18.21	0.99
Chlorogenic acid	-16.23	2.51,2.54,5.28,2.81 2.94	8.31	Lys137,Lys142 Val141,Pro139 Leu137	-18.2	-24.1	-19.20	-19.12	0.98
Harmaline	-10.23	2.73,3.01,2.89,2.85 2.74	10.42	His145,Tyr308 Ser176,Val177 Asp141	-10.21	-20.3	-14.1	-13.23	1.03
Interaction between Riboflavin-5′-monophosphate sodium salt, CHLOROGENIC ACID, and Harmaline	-19.23	2.83,2.88,2.95,2.97 3.06	7.32	Leu137,Val141 Lys142,Tyr146 Asn147	-19.21	-27.2	-20.21	-22.93	0.95
Interaction of Riboflavin-5′-monophosphate sodium salt, CHLOROGENIC ACID, Harmaline with Se	-23.2	2.84,2.73,2.81,2.94,2.86	6.2	Ser144,Tyr146 Asn147,Leu134 Lys142	-20.23	-29.1	-22.29	-25.18	0.91

(a). Riboflavin-5′-monophosphate sodium salt hydrate: The ligand riboflavin-5′-monophosphate resulted in a measured binding energy of − 8.31 kcal/mol and an inhibitory constant (Ki) of 6.23 µM, indicating a moderate affinity to 3GCX. The ligand, both independently and combined, forms four strong hydrogen bonds with Leu134 (1.95 Å), Asn147 (2.84 Å), Tyr146 (2.81 Å), and Ser144 (2.94 Å), interacting with multiple polar contacts of the β-sheet cavity to help stabilize the complex (see first uploaded figure). The overall binding interaction energy properties (ΔG) including electrostatic (–28.43 kcal/mol), van der Waals + H-bond + desolvation energy (–15.2 kcal/mol), and soft constraints of root mean square deviation (RMSD) yielded a ΔG of − 20.3 kcal/mol and RMSD of 0.98 Å for the riboflavin-5′-monophosphate-3GCX complex and provide evidence of structural stability of the 3GCX. (b). Chlorogenic Acid: The compound chlorogenic acid has a stronger affinity (binding energy = − 9.32 kcal/mol; inhibition constant, Ki(µM) = 5.28 µM) via forming dual hydrogen bonds at Lys137 (2.51 Å) and Lys142 (2.54 Å), respectively, while it is also still forming hydrophobic and van der Waals interactions with Val141, Pro139, and Leu137 (see second figure). The electrostatic energy (–29.21 kcal/mol) and total internal energy (–19.3 kcal/mol), allowing for a close orientation of the ligand with a free energy change ΔG = − 21.64 kcal/mol and a root mean square deviation of 0.95 Å, indicate it has a stable anchor position in the same binding groove as riboflavin but has more polarizing interactions.(c). Harmaline: The compound harmaline shows weaker binding (binding energy = − 5.32 kcal/mol; inhibition constant, Ki = 6.92 µM) utilizing Ser147, Glu144, Cys148, Arg68, and Trp112 (third figure). Although harmaline has low electrostatic energy (–21.25 kcal/mol)**, a π–π stacking interaction with Trp112 and direct contact with Arg68 provide stabilization through an aromatic contribution consistent with the rigid structural scaffold of β-carboline.(d). interacted complex (Riboflavin + Chlorogenic + Harmaline): In the end, the multi-ligand complex exhibited a pronounced cooperative binding enhancement (B.E. = -11.37 kcal/mol; Ki = 4.38 µM) shown in the fourth uploaded figure. The presence of several hydrogen bonds that include Asn147 (2.46 Å), Tyr146 (2.61 Å), Ser144 (2.84 Å), Val177 (3.03 Å), Leu137 (2.81 Å), and Glu144 (2.59 Å) form a cooperative network of intermolecular interactions that stabilizes the binding pocket. The ΔG (–22.93 kcal/mol) and RMSD (0.91 Å) values indicated an overall increase in conformational rigidity and lower propensity for dynamic fluctuation compared to individual ligands^97^. (e). Se-chelated complex (Riboflavin, Chlorogenic, Harmaline with Se): The last Se-integrated hybrid complex demonstrated the highest binding affinity (B.E. = -14.32 kcal/mol; Ki(µM) = 4.23 µM). The addition of selenium reinforced the overall π-π and H-bond networks in partnership with Phe146 (2.96 Å), His142 (2.94 Å), Ser144 (2.83 Å), Tyr146 (2.84 Å), as well as aromatic stacking with Trp112, with additional electrostatic binding via Arg 68. The above arrangement exhibited marked electrostatic stabilization (–38.24 kcal/mol) with the lowest ΔG (-25.84 kcal/mol) with RMSD 0.88 Å. Together, the results provided strong evidence suggestive of selenium’s role in improving protein-ligand compactness and bioactive potential (Figure 12A). The of molecular dynamics (MD) simulation provide a summary view of the stability, flexibility, hydrogen-bonding, and contact interactions of the Combined and Se-incorporated 3GCX−ligand complexes over the entire mass of the 100-ns-long trajectory. The plots of root mean square deviation (RMSD) (blue versus orange) shows that both complexes equilibrated quickly during the first 10 ns, where the Se-incorporated complex maintained a tighter deviation from initial structural deviations (approx. 0.88 Å) than the combined complex (0.91 Å). This small reduction in atomic displacement is ascribed to the stabilizing effect of selenium, as the coordination of selenium lichen backbone oscillations while increasing conformational rigidity. The plots of root mean square fluctuation (RMSF) confirmed these data as per-residue fluctuations continued to show lower variability in the Se-incorporated complex particularly among the catalytic pocket residues Asn147, Tyr146, Ser144, and Leu137. This pattern provides evidence that selenium, in conjunction with oxygen, introduced additional coordination constraints to areas of the protein that normally exhibit higher motion, resulting in the stabilization of the hydrogen-bonding network surrounding the active site. The data of hydrogen-bond dynamics reinforces stabilization effects of selenium, where the Se-integrated complex achieved a higher and more consistent number of hydrogen bonds (6–8 bonds) than that of the Combined complex, confirming continued presence of ionic linkages between the phosphate, hydroxide, and indole groups of the ligand and critical residues Tyr146, Ser144, and Arg68.In conclusion, the contact maps display a higher density and persistence of frequency of residue–residue contacts in the Se-integrated system, particularly along the Tyr146–Trp112 and Asn147–Arg68 axes, creating a strong interaction plate that builds binding strength and minimizes solvent access (Fig. 12A). Taken together, the MD profiles show that the Se-integrated complex is both more dynamically stable and provides improved intermolecular interactions, resulting in reduced conformational entropy, providing supporting evidence for the higher docking affinity (ΔG = − 25.84 kcal mol^−1^), and suggesting a selenium bio-synergized configuration, which is bioactive and can stronger inhibitory interaction with HepG2 target 3GCX.

The molecular docking and interaction evaluation of the 5H38 (Anti-cancer) protein with the assessed compounds Riboflavin-5’-monophosphate sodium salt hydrate, Chlorogenic acid, Harmaline, and their combined and Se chelated complexes demonstrates a consistent enhancement in binding affinity and structural stability with integration and the formation of synergy. Riboflavin-5’-monophosphate sodium salt hydrate provides a moderate binding energy with an affinity of -12.29 kcal/mol, through various hydrogen bonds with Leu134, Asn147, Tyr146, and Ser144 measured through distances of 2.81–2.95 Å, and noted stable electrostatic complementarity (ΔG = -18.21 kcal/mol, RMSD = 0.99 Å). Chlorogenic acid provides the highest and best interaction as a stand-alone compound (-16.23 kcal/mol), and showcases five hydrogen and hydrophobic interactions with Lys137, Lys142, Val141, Pro139, and Leu137 at short bond distances between 2.51 and 5.28 Å, and the exhibited polar and nonpolar stabilizing interactions within the active site (ΔG = − 19.12 kcal/mol). Harmaline, while having a lower affinity (-10.23 kcal/mol), demonstrates complementary, π–π, and H-bond interactions with His145, Tyr308, Ser176, Val177, and Asp141, providing evidence of being a co-stabilizing ligand in the mixed complex. When the three ligands acted synergistically in the combined complex, we observed a remarkable enhancement in binding energy (-19.23 kcal/mol) and a lowered RMSD (0.95 Å) compared to the ligands as single agents, indicative of a compact, stable conformation. The cooperative network of residues Leu137, Val141, Lys142, Tyr146, and Asn147 was able to establish dense interaction clusters with bond distances in the range of 2.83–3.06 Å, indicating complementary hydrophobic and polar contacts that are visually supported in the docking figures by the closely-fitting binding pocket and overlapping contours of electron density. Incorporating selenium further improved binding affinity, (-23.2 kcal/mol) with the lowest RMSD (0.91 Å), indicating both a structural rigidity and thermodynamic favorability (ΔG = -25.18 kcal/mol). The Se-integrated complex maintained five stable residues (Ser144, Tyr146, Asn147, Leu134, and Lys142) though the bond distances were shorter (2.73–2.94 Å), suggesting the formation of reinforced Se-mediated coordination and hydrogen bonding, which contributed to electrostatic stabilization in the receptor site (Fig. 12B). The MD simulation of the No-Se and Se-chelated complexes elucidated the propositional structural and energetic differences, showing selenium’s stabilizing role within the 5H38 anti-cancer target system. The RMSD plot showed that No-Se changed between 0.95 and 1.3 Å, whereas the Se-chelated quickly stabilized at a lower RMSD range of 0.85–1.1 Å, indicating better structural stabilization. The RMSF analysis indicated lower flexibility, especially as concerns the catalytic and ligand-binding residues (Leu134, Lys142, Tyr146, Asn147, and Ser144), all pointing to Se coordination as conferring freedom in conformational changes, in turn strengthening the binding pocket. The radius of gyration (Rg) was consistently lower for the Se-chelated complex (~ 2.1 nm) than for the No-Se system (~ 2.3 nm), indicative of a more compact and energetically favorable parent genome. Further, the analysis of hydrogen bonds provided additional evidence that the integration of selenium increased the number of intermolecular H-bonds, stability (average of 8–10 for Se-chelated vs. 6–7 for No-Se), due to imparting slightly more electrostatic stabilization and better solvent interaction. Ultimately, the contact map showed more and wider contact zones in the Se-chelated complex, especially around the active site residue, suggesting greater ligand–protein communication and better binding cooperativity. Taken together, the MD profiles with lower RMSD, smaller RMSF, compact Rg, more hydrogen bonds, and more residue contacts showed that selenium chelation provides a significant reinforcement of conformational rigidity, thermodynamic stability, and bioactive integrity of the tri-ligand system. This is also consistent with the docking-generated free energy (ΔG = − 25.18 kcal/mol) and visual interactions present in Fig. 12B.

### Mechanistic interpretation of docking results

  The detailed mechanistic interpretations regarding the binding assay data, . In addition to the ranking of the three major components identified by LC-MS; i.e. riboflavin-5’-phosphate, chlorogenic acid and harmaline, features of each of these phytochemicals indicate that they engage several key amino acids in the catalytic and structural domains of their target proteins that regulate biological activity.In S. aureus DNA gyrase (PDB: 2XCT), all three phytochemicals created extensive hydrogen-bonding networks to the amino acids Asp141, His145, Ser176, Val177 and Tyr308 that play an integral role in binding to DNA and Mg²⁺-dependent catalysis. Stronger interactions were established with the inclusion of selenium, which resulted in increased occupancy within the active site, as well as extensive π–π and electrostatic stabilization. Perturbation of the active site of S. aureus DNA gyrase invokes an analogue mechanism of action to that previously described for fluoroquinolone antibiotics, supporting the demonstrated antimicrobial properties of SeNPs.In addition, hydrogen-bonding interactions formed to several amino acids within the constriction loop of the porin OmpF (PDB: 3KRA): Asp117, Glu181, Asn182, Ile188 and Tyr177 may provide an alternative mechanistic explanation for the observed inhibition of growth of Gram-negative organisms via disruption of the integrity of their outer membranes and the resultant decrease in membrane permeability and nutrient/ion influx.

For viral proteins (PDBs: 1PAP and 1LP8), selenium-phytochemical assemblies bound to critical amino acids, including Lys197, Ser198, Glu199, Tyr146, and Asn147, forming strong networks of hydrogen bonding which may block or disrupt RNA depurination and/or inhibit viral protein activity. These findings support experimental results indicating that the infectivity of adenovirus and rotavirus was decreased through the actions of these binding interactions. For anticancer targets (PCSK9 and T-HS; PDBs: 3GCX and 5H38), binding regions include Ser144, Tyr146, Asn147, Lys142, plus Leu137, all of which are essential for binding substrates or synthesizing nucleotides. The occurrence of these areas suggests that they could potentially inhibit pathways involved with cholesterol homeostasis or DNA replication, offering additional support for the cytotoxicity demonstrated against HepG2 cells.The biological relevance of the docking interactions was supported by molecular dynamics simulations, which showed stable RMSD values (< 2 Å), persistent hydrogen bonding, and energetically favorable Se-protein complexes over time. While the present study demonstrates promising in vitro antimicrobial, antiviral, and anticancer activity of the biosynthesized SeNPs, the lack of in vivo validation or advanced three-dimensional (3D) cellular models limits direct translational conclusions. Additionally, the percentage yield, batch-to-batch reproducibility, and scalability of the SeNP synthesis were not assessed. Nevertheless, the simple, environmentally friendly, plant-mediated green synthesis approach is inherently amenable to scale-up. Overall, these findings indicate that the phytochemical components of *C. roseus*, when combined with selenium, can form stable complexes capable of interacting with key microbial, viral, and cancer-related proteins. The mechanistic insights provided here support the observed in vitro bioactivities and lay the groundwork for future studies aimed at process optimization, in vivo evaluation, and scale-up production.

## Conclusion

This study demonstrates the green synthesis of selenium nanoparticles (SeNPs) using *Catharanthus roseus* flower extract, with phytochemicals such as riboflavin-5′-phosphate, chlorogenic acid, and harmaline acting as natural reducing and stabilizing agents. The resulting SeNPs were spherical, crystalline (8.6–65.6 nm), and exhibited preliminary bioactivity, including antimicrobial effects, selective antiviral activity against adenovirus, and cytotoxicity against HepG2 liver cancer cells. Molecular docking analyses suggested that these phytochemicals may interact with relevant biological proteins, providing potential mechanistic insights. Importantly, these findings are exploratory, based on in vitro and in silico analyses, and further investigationsincluding the use of additional cancer and normal cell lines, detailed mechanistic studies, and in vivo validationare required to fully evaluate the therapeutic potential and safety of these SeNPs.

## Supplementary Information

Below is the link to the electronic supplementary material.

Supplementary Material 1

## Data Availability

The datasets generated and/or analyzed during the current study are availablefrom the corresponding author on reasonable request. The plant records can often be accessed through online databases and search engines provided by these institutions. For detailed specimen data, visit the Plants of the World Online portal at https://ntbg.org/database/herbarium/detail/PTBG1000042734.
