# Supplementary material for: Phytochemical-mediated green synthesis of selenium nanoparticles using Catharanthus roseus and their physicochemical characterization, biological evaluation, and molecular docking analysis
Source: Sci Rep. 2026 Apr 28;16:13642. doi: 10.1038/s41598-026-47919-3 (PMC13125311; doi:10.1038/s41598-026-47919-3)
Supplement: Supplementary file 1 — Supplementary Material 1 [file 41598_2026_47919_MOESM1_ESM.docx]

**Table (S1):** *LC–MS results for the C. roseus extract* and the role of different extract compounds in SeNP biosynthesis

| Metabolite | Chemical Class | Role in SeNP Synthesis | Mechanistic Notes |
| --- | --- | --- | --- |
| Vinblastine | Alkaloid | Weak reducing potential | Nitrogen heterocycles can coordinate with Se; may enhance stability via complexation. |
| Vincristine | Alkaloid | Weak reducing potential | Nitrogen heterocycles can coordinate with Se; may enhance stability via complexation. |
| Catharanthine | Alkaloid | Weak reducing potential | Nitrogen heterocycles can coordinate with Se; may enhance stability via complexation. |
| Chlorogenic acid | Phenolic acid | Strong reducing and capping agent | Donates electrons to reduce Se⁴⁺ → Se⁰; stabilizes nanoparticles via hydroxyl and carboxyl groups. |
| 3,4-Dimethoxycinnamic acid | Phenolic acid | Moderate reducing agent | Methoxy and carboxyl groups facilitate electron transfer and surface stabilization. |
| TRANS-ORTHO-COUMARIC ACID | Phenolic acid | Capping/stabilizing agent | Aromatic ring and –OH group interact with SeNP surface, preventing aggregation. |
| Kaempferol-3-O-α-L-arabinoside | Flavonoid | Reductant and stabilizer | Polyhydroxylated structure donates electrons and chelates Se atoms; enhances biocompatibility. |
| Quercetin | Flavonoid | Strong reducing agent | Hydroxyl groups (especially 3’,4’-dihydroxy) readily reduce Se ions; also acts as antioxidant shell. |
| Sissotrin | Isoflavonoid | Stabilizing agent | Forms hydrogen bonds and π–π interactions with SeNP surface. |
| Sinapyl alcohol | Phenylpropanoid | Mild reducing agent | Hydroxyl and methoxy groups aid electron donation; supports nanoparticle growth control. |
| Daphnetin / 6,7-Dihydroxycoumarin | Coumarin derivative | Possible stabilizer | Binds to SeNP surface via carbonyl and hydroxyl groups, but less potent reducer. |
| Phytol | Terpenoid alcohol | Hydrophobic stabilizer | Coats nanoparticles to improve dispersion in organic media. |
| Riboflavin-5′-monophosphate | Vitamin (phenolic-like) | Weak electron donor | May contribute to reduction via isoalloxazine ring but mainly acts as stabilizer. |
| Harmaline | Alkaloid | Weak reducing potential | Nitrogen heterocycles can coordinate with Se; may enhance stability via complexation. |

Summary of Roles:

• Major reducing agents: Vinblastine, Vincristine, Catharanthine, Chlorogenic acid, Quercetin, Kaempferol-3-O-α-L-arabinoside.

• Major stabilizers: Sissotrin, Coumaric acid, Coumarins, Phytol.

• Minor contributors: Harmaline and Riboflavin-5′-monophosphate act as mild stabilizers

**Table (S2):** Comparison of antibacterial, antifungal, MIC, and MBC values of green-synthesized SeNPs in the present study with previously reported plant-based SeNPs.

| Study / Source of SeNPs | Microorganisms Tested | Inhibition Zone (mm) | MIC (µg/mL) | MBC (µg/mL) | Key Notes |
| --- | --- | --- | --- | --- | --- |
| Present study (C. roseus–SeNPs) | *B. subtilis*, *S. aureus*, *E. coli*, *P. aeruginosa*, *C. albicans* | **32–35 (Gram+)**, 16–25 (Gram–), **30 (fungus)** | **10.5–26.25** | **420–636.36** | Higher activity than extract and commercial antibiotics; strong ROS-mediated effects |
| *Aloe vera*–SeNPs (Khurana et al., 2024) | *E. coli*, *S. aureus*, *C. albicans* | 14–22 | 50–125 | 500–1000 | Moderate antibacterial; limited antifungal effect |
| *Camellia sinensis*–SeNPs (Alizadeh et al., 2023) | Gram+ and Gram– bacteria | 12–25 | 25–200 | 600–1000 | Activity varies with particle size; moderate ROS induction |
| *Allium sativum*–SeNPs (Puri et al., 2024) | *S. aureus*, *E. coli* | 15–28 | 30–150 | 500–900 | Stronger against Gram+; phytochemicals enhance stability |
| *Withania somnifera*–SeNPs (Li et al., 2025b) | *S. aureus*, *P. aeruginosa*, *C. albicans* | 12–20 | 40–200 | 700–1200 | Lower activity due to larger particle size |
| *Aloe barbadensis*–SeNPs (Sans-Serramitjana et al., 2023) | Multiple bacterial strains | 13–18 | 60–175 | 700–1000 | ROS-mediated antibacterial effect; weaker than current study |

**Table (S3):** Comparison of antiviral activity of selenium nanoparticles (SeNPs) from the current study with previously reported SeNPs antiviral results.

| Study / SeNP Source | Virus Tested | Antiviral Indicator | Results / Measurement | Notes |
| --- | --- | --- | --- | --- |
| Present study (C. roseus–SeNPs) | Adenovirus | IC50 (μg/mL) | **22.99 μg/mL** | Significant cytotoxic effect and measurable IC50 against adenovirus |
| Present study | Rotavirus | Antiviral effect | No significant inhibition | Likely due to high rotavirus resistance |
| *Polycladia myrica–SeNPs* (brown algae) | HAV-10, Adenovirus, HSV‑2 | % Inhibition at 125 μg/mL | 40.25% (HAV‑10), 8.64% (Adenovirus), 17.39% (HSV‑2) | Demonstrated moderate antiviral activity; adenovirus effect weaker than for HAV |
| *Se@TP functionalized SeNPs* | H1N1 influenza | % Cell viability increase / viral inhibition | Not quantified in same units; protective effect via reduced apoptosis | Functionalized SeNPs improved antiviral activity and cell survival |
| PEG‑SeNPs (recent study) | H1N1 influenza | Antiviral activity / reduced apoptosis | Protective effect on infected MDCK cell | Shows antiviral and anti‑inflammatory effects |
| Review summary (various SeNPs) | EV71 / H1N1 / HBV etc. | General antiviral effects | Broad evidence of antiviral potential across viruses | Provides context for diverse SeNP antiviral responses |

**Table (S4).** Comparative cytotoxicity of green-synthesized SeNPs on HepG2 and other cancer cell lines.

| Study / SeNP Source | Cell Line Tested | IC50 (μg/mL) | Notes / Key Findings |
| --- | --- | --- | --- |
| Present study (C. roseus–SeNPs) | HepG2 | **1.5** | Strong cytotoxic effect; clear dose-response; potent plant-mediated effect |
| Krishnan et al., 2019 | HepG2 | 30 | Biosynthesized SeNPs showed moderate inhibition |
| Cui et al., 2018 (hawthorn fruit) | HepG2 | 19.22 | Induced apoptosis; moderate cytotoxicity |
| Al-Duais et al., 2025 | MCF-7, A549 | 10–25 | Green SeNPs showed selective cytotoxicity against breast and lung cancer cells |
| Ahmed et al., 2025 | HeLa | 12 | Strong anticancer effect due to phytochemical capping agents |

**Figure (1 S):** IC50 (μg/ml) for SeNP biosynthesized by C. roseus extract

**Figure 2S:** Anti- HepG2 cells activity of the SeNPs biosynthesized through C. roseus extract
